# The role of osmolarity adjusting agents in the regulation of encapsulated cell behavior to provide a safer and more predictable delivery of therapeutics

**DOI:** 10.1080/10717544.2017.1391894

**Published:** 2017-10-27

**Authors:** Ainhoa Gonzalez-Pujana, Aitor Rementeria, Francisco Javier Blanco, Manoli Igartua, Jose Luis Pedraz, Edorta Santos-Vizcaino, Rosa Maria Hernandez

**Affiliations:** a NanoBioCel Group, Laboratory of Pharmaceutics, School of Pharmacy, University of the Basque Country (UPV/EHU), Vitoria-Gasteiz, Spain;; b Biomedical Research Networking Centre in Bioengineering, Biomaterials and Nanomedicine (CIBER-BBN), Vitoria-Gasteiz, Spain;; c Department of Immunology, Microbiology and Parasitology, Faculty of Science and Technology, Fungal and Bacterial Biomics Research Group, University of the Basque Country (UPV/EHU), Leioa, Spain;; d CIBER-BBN-Bioscaff Cartílago, INIBIC Institute for Biomedical research, A Coruña, Spain

**Keywords:** Drug delivery, cell therapy, microcapsule, hydrogel, alginate

## Abstract

Transplantation of cells within alginate microspheres has been extensively studied for sustained drug delivery. However, the lack of control over cell behavior represents a major concern regarding the efficacy and the safety of the therapy. Here, we demonstrated that when formulating the biosystem, an adequate selection of osmolarity adjusting agents significantly contributes to the regulation of cell responses. Our data showed that these agents interact in the capsule formation process, influencing the alginate crosslinking degree. Therefore, when selecting inert or electrolyte-based osmolarity adjusting agents to encapsulate D1 multipotent mesenchymal stromal cells (MSCs), alginate microcapsules with differing mechanical properties were obtained. Since mechanical forces acting on cells influence their behavior, contrasting cell responses were observed both, *in vitro* and *in vivo*. When employing mannitol as an inert osmolarity adjusting agent, microcapsules presented a more permissive matrix, allowing a tumoral-like behavior. This resulted in the formation of enormous cell-aggregates that presented necrotic cores and protruding peripheral cells, rendering the therapy unpredictable, dysfunctional, and unsafe. Conversely, the use of electrolyte osmolarity adjusting agents, including calcium or sodium, provided the capsule with a suitable crosslinking degree that established a tight control over cell proliferation and enabled an adequate therapeutic regimen *in vivo*. The crucial impact of these agents was confirmed when gene expression studies reported pivotal divergences not only in proliferative pathways, but also in genes involved in survival, migration, and differentiation. Altogether, our results prove osmolarity adjusting agents as an effective tool to regulate cell behavior and obtain safer and more predictable therapies.

## 1. Introduction

The entrapment of therapeutically active cells within alginate microspheres has been widely employed for the sustained delivery of therapeutics (Strand et al., 2017). The long-term function of the system allows the continuous production of therapeutics, avoiding the necessity of frequent administrations and hence, improving patient’s quality of life. However, the performance of the system is directly dependent on cell responses and nowadays implant functionality and safety are still importantly limited by a lack of control over cell behavior (Santos et al., 2013). Among the erratic cell responses, excessive proliferation rates represent one of the major hurdles, especially when it comes to using immortalized cell lines (Bhujbal et al., 2014). Cellular overgrowth results in aggregates that present a characteristic dualism. On the one hand, the limited diffusion of nutrients and oxygen to the core of the aggregates results in inner cell death (de Vos, 2017). This does not only lead to a diminished therapeutic effect, but has additional consequences, since intracellular components derived from dying cells, namely danger-associated molecular patterns (DAMPs), function as alarm molecules that evoke important immune and inflammatory responses (Paredes Juarez et al., 2014). On the other hand, protruding peripheral cells may leak out of the matrix, constituting a major safety concern (Bhujbal et al., 2014).

In attempting to develop systems with improved control over cell behavior, tuning the mechanical properties of the matrix has been suggested as a valuable strategy. This is possible because of the mechanotransduction process, by which mechanical forces acting on cells influence their biochemical behavior and viability (Humphrey et al., 2014). Taking advantage of it, we focused on osmolarity adjusting agents as a tool to modify the mechanics of the capsule and therefore, regulate cell behavior. The process of cell encapsulation requires the dissolution of the employed biomaterials. Therefore, to meet the standards of cell culture, the solutions of, e.g. alginate or poly-L-lysine (PLL) should present physiological osmolarity values between 260 and 320 mOsm/L (Ozturk & Palsson, 1991). To this end, osmolarity adjusting agents are included in the solutions, which can be classified as electrolyte or inert agents. Considering that microspheres are ionically crosslinked matrices, the presence or absence of electrolytes, especially divalent cations, may influence the capsule formation process. Therefore, the use of different types of osmolarity adjusting agents may alter the mechanical properties of the matrix, having an impact on cell responses. To the best of our knowledge, the effect of these agents in cell behavior and consequently, in the outcome of the therapy, has not been previously studied in depth.

Here, we designed two sets of solutions (each one including all the solutions required for the elaboration of microcapsules: 1.5% alginate, 0.05% PLL, 0.1% alginate and washing solutions), which differed in the selected type of osmolarity adjusting agent. The biological set contained electrolytes including calcium, sodium, or phosphates and the technological was based on mannitol as an inert agent. These solutions were used to encapsulate D1 multipotent mesenchymal stromal cells (MSCs). MSCs were genetically modified to express erythropoietin (EPO), a model therapeutic molecule that can be easily traced *in vivo* through hematocrit measurements to assess the functionality of the implant. The resulting biological and technological microcapsules were characterized and our data showed that the distinct mechanical properties of the matrix influenced cells at a genic level. This resulted in a contrasting cell behavior, which led to divergent therapeutic profiles *in vitro* and *in vivo*, highlighting the pivotal importance of an accurate formulation to obtain systems with suitable properties that control cell behavior.

## 2. Methods

### 2.1. Characterization of the solutions

For characterization, pH was determined by means of the pH-Meter GLP 21 (Crison^®^, Barcelona, Spain). Osmotic pressure was assessed by using a cryoscopic osmometer (Osmomat 030, Gonotec^®^, Berlin,Germany). This type of osmometer measures the freezing point depression, which is directly proportional to the concentration of osmotically active compounds in aqueous solutions. To calibrate the osmometer, NaCl calibrating solution was employed. Prior to the measurement, its osmolarity value was set in the osmometer. Subsequently, 50 µL of the calibrating solution were pipetted into a clean, dry measuring vessel. After ensuring there were no visible air bubbles, the measurement was performed and the result was automatically adopted as the calibration value. Next, sample measurements were performed under the same conditions. Each sample was assayed in triplicate.

### 2.2. Cell culture

MSCs (ATCC^®^ CRL 12424TM, Manassas, VA) were genetically modified with the lentiviral vector pSIN-EF2-*Epo*-Pur to express EPO (D1-MSCs-EPO) (Gurruchaga et al., 2015). Cells were seeded in T-flasks and grown in Dulbecco’s modified Eagle’s medium (DMEM) supplemented with 10% (v/v) fetal bovine serum (FBS), and 1% (v/v) penicillin/streptomycin. They were maintained at 37 °C in a 5% CO_2_/95% air atmosphere and passaged every 2–3 d using trypsin-EDTA. All reagents were purchased from Fisher Scientific, Madrid, Spain.

### 2.3. Cell microencapsulation

D1-MSCs-EPO were encapsulated using an electrostatic droplet generator (Nisco^®^, Duluth, GA), following the procedure designed by Lim and Sun (Lim & Sun, 1980). Briefly, cells were suspended in 1.5% (w/v) sodium alginate obtaining a cell density of 5 × 10^6^ cells/mL. This suspension was extruded through a 0.35 mm needle at a 5.9 mL/h flow rate by means of a peristaltic pump. Beads were collected in a 100 mM CaCl_2_ bath and maintained in agitation for 10 min to ensure a complete ionic gelation. After washing the particles, they were suspended in 0.05% (w/v) PLL for 5 min. Once washed, a second coating was performed by suspending the particles in 0.1% (w/v) alginate for 5 min, giving rise to alginate-poly-L-lysine-alginate (APA) microcapsules. Since different solutions of 1.5% alginate, 0.05% PLL, 0.1% alginate and washings were designed, three types of APA microcapsules were obtained: Biological microcapsules (made of biological solutions), technological microcapsules (made of technological solutions), and hybrid microcapsules (made of biological 1.5% alginate and technological 0.05% PLL, 0.1% alginate and washing solutions) (Supplementary Figure S1). This last group was intended for the structural studies, to determine the influence of core and coating solutions separately in the final properties of the capsule. Regarding the 100 mM CaCl_2_ bath, the same solution was employed for every group, since the presence of the gelling ion Ca^+2^ was mandatory. Microcapsules were cultured in complete medium at 37 °C in a 5% CO_2_/95% air atmosphere. All the process was carried out under aseptic conditions at room temperature. Ultra-pure low-viscosity high guluronic acid alginate (UPLVG, code #4200006) was purchased from FMC Biopolymer, Sandvika, Norway and PLL (hydrobromide Mw 15,000–30,000 Da, code P7890-500MG) from Sigma-Aldrich, Madrid, Spain.

### 2.4. Cell aggregate area quantification

Micrographs were obtained by an inverted microscope (Nikon TMS, Hampton, NH) and processed by means of Image*J* software (Rasband, W.S., ImageJ, US National Institutes of Health, Bethesda, Rockville, MD, https://imagej.nih.gov/ij/, 1997–2016) to quantify the cell-aggregate area. For each time point, aggregates of at least 20 random capsules were analyzed.

### 2.5. Cell proliferation: bromodeoxyuridine (BrdU) uptake

BrdU uptake was determined by means of Cell Proliferation Biotrak ELISA System (code GERPN250, Sigma Aldrich, Madrid, Spain) in five independent samples per group. In a 96 well-plate, the equivalent of 2 × 10^4^ cells was placed in each well (≈80 microcapsules). All groups were incubated for 24 h in complete medium supplemented with 10% FBS, except for the starving control group, which was incubated in medium supplemented with 0.1% FBS. An additional 24-h incubation was carried out in presence of 10 μM BrdU, except for the nonspecific binding control group, in which the reagent was not added. Subsequently, cells were de-encapsulated by means of a 500 μg/mL solution of alginate lyase (code A1603, Sigma-Aldrich, Madrid, Spain) and the assay was performed following manufacturer’s indications. Absorbance measurements were normalized with the nonspecific binding control.

### 2.6. Viability assays

Three different assays were performed to evaluate the viability of the immobilized cells: live/dead staining, flow cytometry, and cell metabolism studies. For live/dead staining 10 µL of microcapsules containing D1-MSCs-EPO were dyed with the LIVE/DEAD^®^ kit (code L3224, Fisher Scientific, Madrid, Spain) following manufacture’s indications. After 30 min, fluorescence micrographs were taken using an epi-fluorescence microscope (Nikon TSM, Hampton, NH). At least six independent experiments were analyzed for each group.

For flow cytometry (BD FACS Calibur, San Jose, CA), cells were de-encapsulated using a 500 μg/mL solution of alginate lyase. Cells were then treated with trypsin-EDTA to eliminate possible cell-aggregates and dyed with the LIVE/DEAD^®^ kit. After 20-min incubation, protected from light and at room temperature, cells were acquired. Three independent samples of cells from each group were assayed.

Metabolic activity was determined using the Cell Counting Kit-8 (CCK-8) (code 96992-3000TESTS-F, Sigma Aldrich, Madrid, Spain). Approximately 50 microcapsules (≈ 12,300 cells) were suspended in 100 μL of DMEM and placed in a 96-well plate. Subsequently, 10 μL of CCK-8 were added to each well. Plates were incubated for 4 h at 37 °C and read on an Infinite M200 TECAN plate reader (GMI Inc., Ramsey, MN) at 450 nm, with reference wavelength at 650 nm. At least seven independent experiments were analyzed for each group.

### 2.7. EPO secretion

EPO secretion was determined by means of the Quantikine IVD Human EPO ELISA Kit (code DEP00, R&D Systems, Madrid, Spain). 100 μL of microcapsules were incubated for 24 h at 37 °C and supernatants were assayed. Samples and standards were run in duplicate following manufacturer’s instructions. Per study group three independent samples were assayed.

### 2.8. Diameter determination and osmotic resistance test

Micrographs were obtained by an inverted microscope and analyzed using the Image*J* software. To evaluate osmotic resistance 100 μL of microcapsules were suspended in 1 mL of deionized water (ddH_2_O) and placed in 12 well plates. After 5 min, supernatants were replaced with fresh ddH_2_O to perform a second washing. The process was repeated to a total of five washings. Micrographs were taken previous to the assay and after each washing to determine diameter and integrity of the capsules. At least 30 microcapsules were analyzed in both assays.

### 2.9. Fluorescein isothiocyanate (FITC)-dextran diffusion

In order to determine the microcapsule membrane molecular weight cutoff (MWCO), FITC-dextrans were employed (Mw 10, 20, 40, 70, and 150 kDa). A volume of 10 μL of microcapsule suspension (≈170 capsules) was washed and a 0.5 mg/mL FITC-dextran solution was added. Samples were incubated at room temperature for 24 h and observed by confocal microscopy (Leica TCS SP2 AOBS Spectral Confocal Scanner (Buffalo, NY) mounted on a Leica DM IRE2 inverted fluorescent microscope, Wetzlar, Germany). Micrographs were analyzed using Image*J*. Equal area squares were defined and the relative intensity of 20 microcapsules and 20 background areas was determined to obtain the dextran diffusion percentage. Per study group four independent samples were assayed.

### 2.10. Crosslinking ion determination

Microcapsules with no cell load were treated with a 500 μg/mL solution of alginate lyase in order to cause their rupture and release all the calcium forming the matrix. A colorimetric Calcium Detection Kit (code ab102505, Abcam, Barcelona, Spain) was employed to perform the calcium determination. Each group was assayed in triplicate.

### 2.11. Cell cycle

In this method, BrdU (an analogous of the DNA precursor thymidine) was incorporated into newly synthesized DNA. Additionally, cells were dyed with 7-aminoactinomycin D (7-AAD), which binds to the total DNA and resolves cell cycle phases in our populations (G0/1 (resting phase) or S/G2/M (DNA synthesis and division). With that purpose, cells were previously de-encapsulated using a 500 μg/mL solution of alginate lyase and treated using the BrdU Flow Kit Staining Protocol (code 559619, BD Biosciences, Madrid, Spain). Finally, three independent samples from each group were analyzed by flow cytometry.

### 2.12. Animal experimentation

*In vivo* studies were performed according to the ethical guidelines established by the institutional animal care and use committee of the University of Basque Country UPV/EHU (Permit number: CEEA_411_2015_HERNÁNDEZ MARTÍN). Fifteen female 6-week-old C57BL/6 mice were chosen as allogenic immunocompetent murine models (*n* = 5 per group: biological, technological, and control). Animals were anesthesised by isofluorane inhalation and subcutaneously implanted with a total volume of 60 µL of microcapsules (suspended in additional 300 µL of Dulbecco's Phosphate-Buffered Saline (DPBS) code BE17-513F, Lonza, O Porriño, Spain) by means of a 20-gauge catheter. For the control group, sole DPBS was administered. At days 15, 30, and 45 blood samples were collected in heparinized capillary tubes by facial vein puncture. The obtained whole blood was centrifuged at 760 × *g* for 15 min and hematocrit levels were determined using a standard microhematocrit method. At day 45 mice were sacrificed. Samples were retrieved and fixed in 4% paraformaldehyde for histological analysis. Hematoxylin and eosin staining was performed and results were blindly evaluated by a pathologist.

### 2.13. RNA isolation, microarray hybridization, and transcriptomic analysis

Total RNA was extracted from four independent samples of cells within biological or technological microcapsules, using Tri Reagent solution (Fisher Scientific, Madrid, Spain), and the modified precipitation protocol recommended for sources rich in polysaccharides and proteoglycans. After the addition of 10 µg of glycogen, RNA was further purified with PureLink RNA Mini Kit (Fisher Scientific, Madrid, Spain). RNA concentration and purity were assessed by NanoDrop 1000 spectrophotometer (NanoDrop Technologies Inc.,Wilmington, DE) and RNA quality and integrity were assayed by Lab-chip technology on an Agilent 2100 Bioanalyzer with Agilent RNA 6000 Nano Chips (Agilent technologies, Santa Clara, CA). All obtained RNA samples were degraded with RNA integrity number (RIN) values 1–2. Whole mouse gene expression microarray analysis was performed using the available SurePrint G3 Mouse GE v2 8x60K Microarray (Agilent Microarray Design ID: ID 074809, Agilent Technologies, Santa Clara, CA). Nucleic acid from each replicate, 100 ng, were labeled following the Agilent protocol “Gene Expression FFPE Workflow”. Feature Extraction Software version 10.7.3.1 (Agilent Technologies, Santa Clara, CA) was used to convert the image into expression data. Raw data were preprocessed, normalized and filtered using GeneSpring GX version 13.0 software (Agilent Technologies, Santa Clara, CA). Data were normalized with quantile method and filtered based on coefficient of variation (CV <100%), obtaining the log_2_ of the average value of signal intensity for each probe. LIMMA statistical package (Smyth, 2004) from the Multi Experiment Viewer (MEV) software version 4.9 (GraphPad Software Inc, La Jolla, CA) (Saeed et al., 2006) was used for differential gene expression analysis. Conventional statistical criteria (adjusted *p* value <.05) were used for the selection of differentially expressed genes. Microarray data have been deposited in the ArrayExpress database at EMBL-EBI (www.ebi.ac.uk/arrayexpress) under accession number E-MTAB-5786. (For further details, see the supplementary data).

### 2.14. Data analysis and statistics

To detect significant differences between two groups Student’s t-test was used, while one-way ANOVA was chosen for multiple comparisons. Depending on the results of the Levene test of homogeneity of variances, Bonferroni or Tamhane post-hoc test was applied. For non-normally distributed data, Mann-Whitney nonparametric analysis was applied. All statistical computations were performed by SPSS 23 (IBM SPSS, Chicago, IL). Data are shown as mean ± SD.

## 3. Results

### 3.1 Characterization of the solutions

To determine the influence of selecting inert or electrolyte osmolarity adjusting agents, two sets of solutions were designed: a biological set (containing electrolytes as osmolarity adjusting agents) and a technological set (with mannitol as an inert agent). During the design, preliminary experiments were performed to determine the best vehicle containing electrolytes for the biological 1.5% alginate solution. Among DMEM w/o Ca^+2^ and Mg^+2^, hanks balanced salt solution (HBSS) and phosphate buffered saline (PBS), the former was selected. Despite presenting osmolarity values slightly above the limits, it was chosen for providing the best results regarding cell viability and metabolic activity (Supplementary Figure S2). The resulting sets were characterized (Table 1) showing physiological pH and osmolarity values.

**Table 1.** Characterization of the biological and technological sets of solutions. Composition, pH, and osmolarity (mOsm/L).

Solution	Composition	pH	Osmolarity (mOsm/L)
Biological set			
1.5% Alginate	1.5% alginate, DMEM w/o Ca^2+^, Mg^2+^	8.01 ± 0.30	345 ± 17
0.05% PLL	0.05% PLL, DPBS w/Ca^2+^, Mg^2+^	7.23 ± 0.05	277 ± 14
0.1% Alginate	0.1% alginate, DPBS w/Ca^2+^, Mg^2+^	7.12 ± 0.04	282 ± 16
100 mM CaCl_2_	100 mM CaCl_2_, 25 mM HEPES buffer, 0.7% mannitol, ddH_2_O	7.04 ± 0.05	284 ± 17
Washings	CaCl_2_ to 2.5 mM calcium, DPBS w/Ca^2+^, Mg^2+^	7.13 ± 0.04	286 ± 06
Technological set			
1.5% Alginate	1.5% alginate, 25 mM HEPES buffer, 4.3% mannitol, ddH_2_O	7.22 ± 0.16	305 ± 07
0.05% PLL	0.05% PLL, 25 mM HEPES buffer, 4.7% mannitol, ddH_2_O	7.20 ± 0.10	289 ± 11
0.1% Alginate	0.1% alginate, 25 mM HEPES buffer, 4.7% mannitol, ddH_2_O	7.09 ± 0.04	306 ± 04
100 mM CaCl_2_	100 mM CaCl_2_, 25 mM HEPES buffer, 0.7% mannitol, ddH_2_O	7.04 ± 0.05	284 ± 17
1° Washing	CaCl_2_ to 2.5 mM calcium, 25 mM HEPES buffer, 4.5% mannitol, ddH_2_O	7.06 ± 0.10	292 ± 03
Rest of washings	25 mM HEPES buffer, 4.8% mannitol, ddH_2_O	7.10 ± 0.02	292 ± 06

Each value represents mean ± SD (*n*** **= 3). DMEM: Dulbecco’s modified Eagle’s medium; PLL: poly-L-Lysine; DPBS: Dulbecco's phosphate-buffered saline; HEPES – N′-2: hydroxyethylpiperazine-N′-2 ethanesulphonic acid; ddH_2_O: deionized water.

### 3.2. APA microcapsules formulated with different types of osmolarity adjusting agents led to contrasting cell behavior *in vitro*

**Figure 1.**
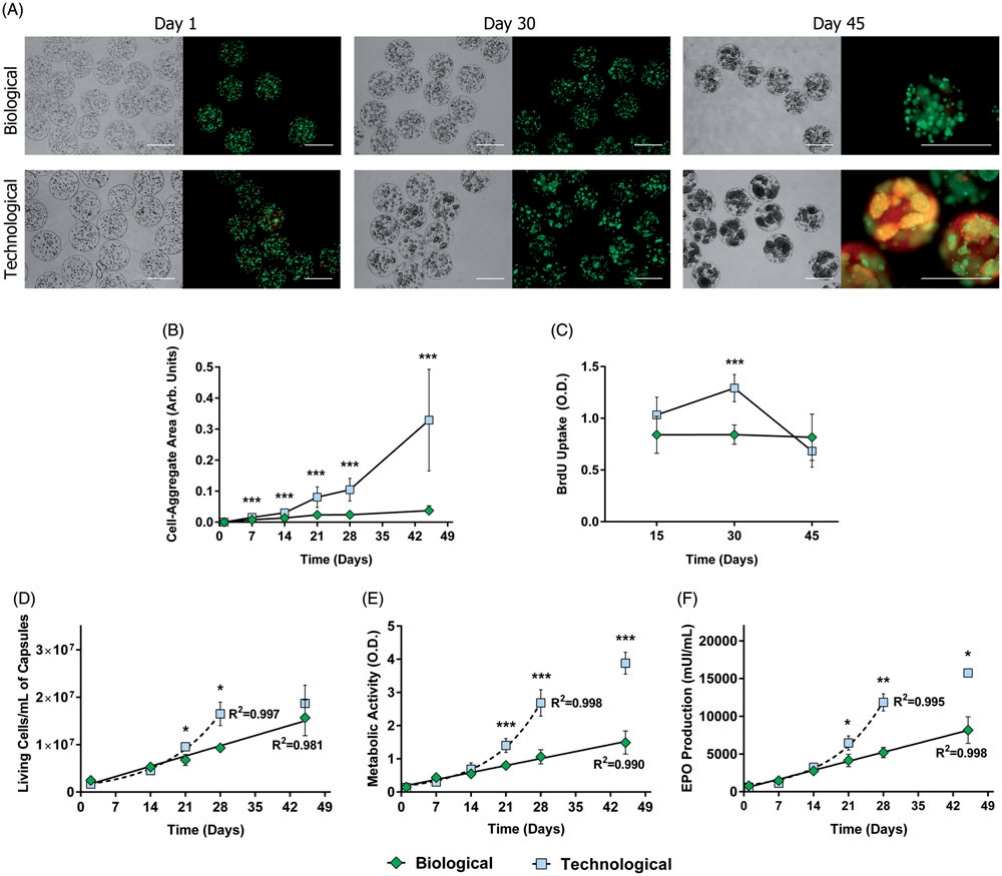
APA microcapsules formulated with different types of osmolarity adjusting agents led to contrasting cell behavior in vitro. (A) Bright field and LIVE/DEAD fluorescence micrographs taken at different time points after cell encapsulation revealed diverging cell behavior. (B) Subsequent quantification of cell-aggregate areas demonstrated statistically significant differences. (C) Bromodeoxyuridine (BrdU) uptake studies confirmed these data by showing an important increase in proliferation in the technological group by day 30. Consequently, the biological biosystem presented a linear tendency for cell viability (D), metabolic activity (E), and erythropoietin (EPO) production (F), whereas technological capsules followed an exponential trend up to day 30. From that point on, this group withdrew from its tendency due to an increase in cell death. Line graphs symbolize mean ± SD (*n* = 5 for BrdU uptake, *n* = 3 for cell viability, and EPO production studies, *n* = 7 for metabolic assays). Statistical significance: *p < .05, **p < .01 and ***p < .001. Scale bars = 400 µm.

The biological and technological sets of solutions were employed to encapsulate D1-MSCs-EPO, obtaining biological and technological microcapsules, respectively. In order to study cell behavior within each group, microcapsules were cultured *in vitro* for 45 d. During the course of the study, important cell-aggregates started to emerge in technological microcapsules, fact that was not so evident in the biological group (Figure 1(A)). Their area was quantified confirming significant differences from day 21 (*p* < .001) (Figure 1(B)). BrdU uptake assays supported these data by demonstrating that the proliferation rate remained constant in the biological group whereas it showed important variations for technological microcapsules.

In particular, for the latter, cell division significantly increased by day 30 (*p* < .001) but drastically decreased by day 45 (Figure 1(C)). This drop at the end of the study may be originated by reduced cell viability, as shown in calcein/ethidium fluorescent micrographs (Figure 1(A)). This phenomenon was also observed in flow cytometry analysis (Figure 1(D)). Although the number of viable cells per mL of capsules increased in both groups over the study, Biological microcapsules adjusted to a linear tendency (*R*^2^ = 0.981), whereas the technological group followed an exponential one (*R*^2^ = 0.997) (Figure 1(D)). Nevertheless, by day 45, the latter withdrew from its trend, pointing out, again, to increased cell death. The exact same profiles were obtained for cell metabolism (Figure 1(E)) and therapeutic factor production (Figure 1(F)).

### 3.3. Mechanical studies revealed relevant differences in the structural properties

**Figure 2.**
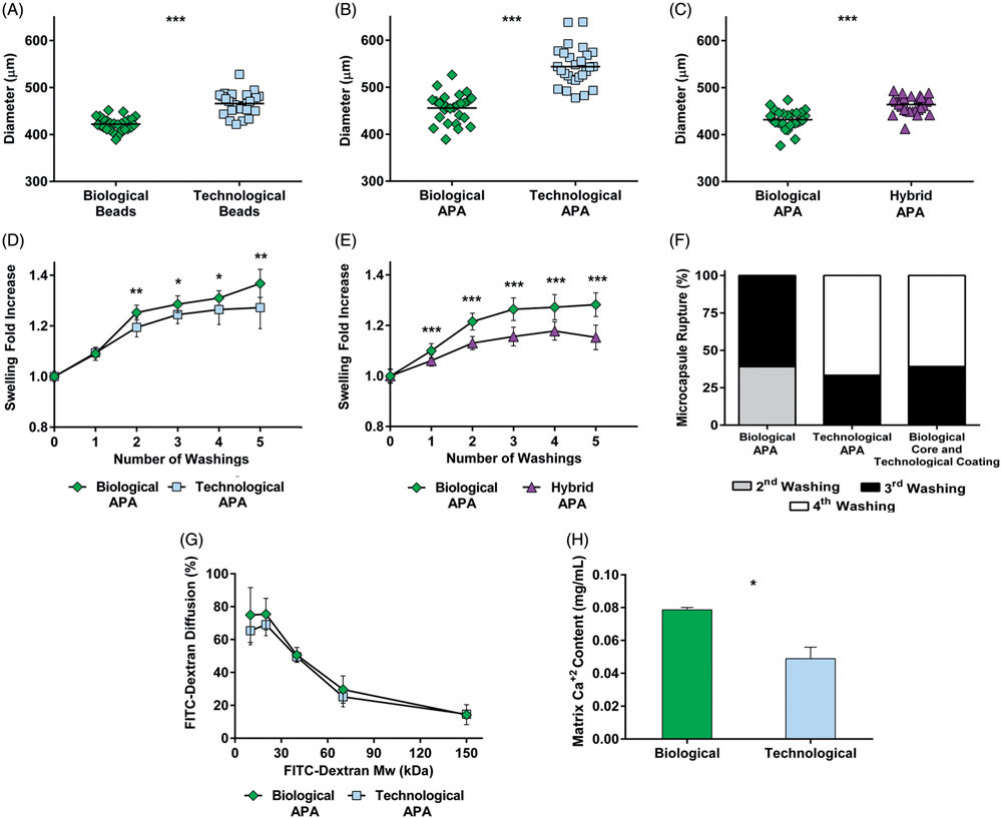
Mechanical studies revealed important differences in the structural properties of microcapsules. Diameter quantification of uncoated beads (A), alginate-poly-L-lysine-alginate (APA) microcapsules (B) and a comparison between biological and hybrid microcapsules (biological core and technological coatings) (C). In all cases, greater sizes were observed when employing technological solutions. Osmotic resistance was assessed for APA microcapsules (D) and a comparison between biological and hybrid microcapsules (E) and both showed an increased swelling behavior for the biological group. (F) Despite the three types of microcapsules presented a good resistance to rupture, it was enhanced in the hybrid and technological groups. (G) FITC labeled dextran diffusion showed no differences in permeability. (H) Calcium release assays proved a higher crosslinking degree in biological matrices. Graphs symbolize mean ± SD (*n* = 30 for diameter quantification and osmotic resistance, *n* = 3 for calcium determination assays, *n* = 4 for FITC dextran diffusion). Statistical significance: *p < .05, **p <.01, and ***p < .001.

To determine if the incorporation of different types of osmolarity adjusting agents influenced the configuration of the capsule, mechanical studies were performed. When comparing biological versus technological beads (microspheres with no coatings), larger diameters were observed in the latter (*p* < .001) (Figure 2(A)). Subsequently, the diameters of coated microcapsules (APA microcapsules) were analyzed, obtaining, again, higher values for the technological group (*p* < .001) (Figure 2(B)). In order to elucidate if the increase in size was only due to the core solution (1.5% alginate) or on the contrary, to a synergistic effect between core and coating solutions (0.05% PLL and 0.1% alginate), biological microcapsules were compared to hybrid capsules (biological core and technological coatings). Hybrid particles presented larger size (*p* < .001) (Figure 2(C)) confirming the direct effect of both core and coating solutions in the size of the capsule.

We next evaluated the osmotic resistance by performing ddH_2_O washings. Higher swelling values were observed in the biological group when compared to technological (*p* < .05) (Figure 2(D)) or hybrid microcapsules (*p* < .001) (Figure 2(E)). The number of broken capsules after each washing was also quantified (Figure 2(F)). For the biological group, a 40% of the microcapsules broke in the second washing, while the rest exploded in the third. Contrarily, both technological and hybrid microcapsules presented a slightly higher resistance: around a 40% exploded in the third washing, and the remaining did it in the fourth. Concerning permeability, confocal fluorescent micrographs (Supplementary Figure S3) revealed that the FITC-dextran diffusion presented no statistical differences in the MWCO (Figure 2(G)). To finish with the mechanical characterization, the calcium-mediated crosslinking of the alginate matrix was analyzed. A significantly higher release of calcium from biological matrices (*p* < .05) suggested they contained a greater concentration of the divalent ion, and consequently, a higher crosslinking degree (Figure 2(H)).

### 3.4. Cell cycle analysis demonstrated that the mechanical differences led to differing cell behavior *in vitro*

**Figure 3.**
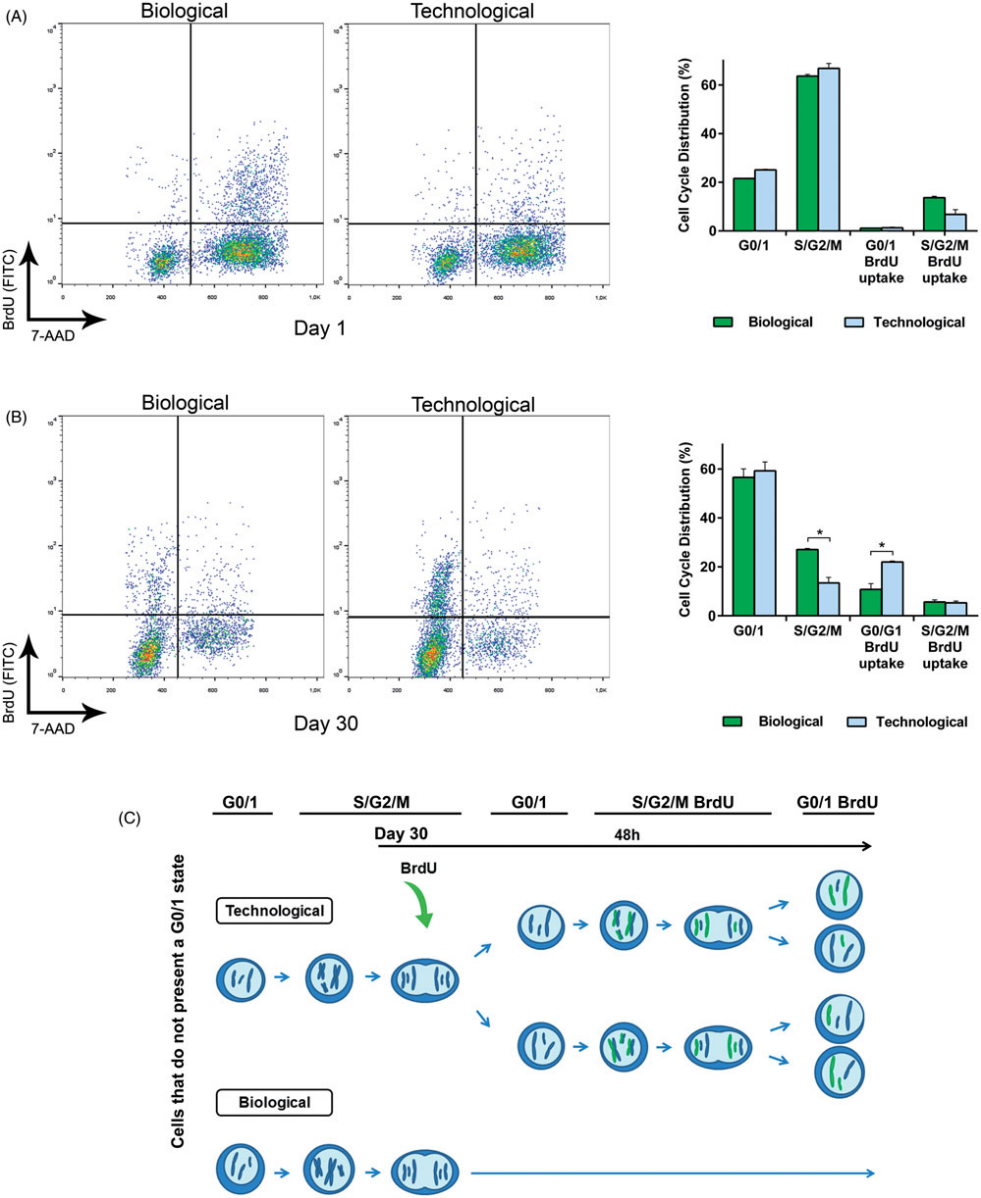
Cell cycle analysis demonstrated that the mechanical differences led to differing cell behavior in vitro. (A) Flow cytometry combining bromodeoxyuridine (BrdU) uptake and 7-aminoactinomycin D (7-AAD) staining showed no statistically significant differences at the very beginning of the study. (B) This tendency dramatically changed by day 30, where the proliferation rate of the technological group significantly increased. (C) Schematic representation of the results obtained in the combined flow cytometry assay at day 30. In particular, the scheme shows the proliferative capacity of the remaining cells that do not present a G0/1 state (44.7% biological, 42.1% technological). Graphs symbolize mean ± SD (*n* = 3). Statistical significance: *p < .05.

Since the mechanical characterization of microcapsules determined a higher crosslinking degree in the biological matrix, we hypothesized this may be the factor which controlled the aggregate formation in this group. To shed light on this issue, we studied simultaneously cell cycle and proliferation. At day 1 after encapsulation, the majority of cells in both groups presented an S/G2/M state with no BrdU uptake (63.3% for biological and 66.1% for technological) (Figure 3(A)). According to these results, before the addition of BrdU cells had already duplicated their genetic material and invested the following 48 h, in presence of BrdU, in the step previous to cell division.

At day 30, the majority of cells in both groups presented a G0/1 state with no BdrU uptake (55.3% for biological and 57.9% for technological) (Figure 3(B)). However, interesting differences were observed in the remaining states. The biological group followed a similar pattern in comparison to day 1, having the highest percentage of those remaining cells in S/G2/M state with no BrdU uptake. Nevertheless, the trend changed for the technological group. The majority of the remaining cells presented a G0/1 state with BrdU uptake. Therefore, after the addition of BrdU cells duplicated their genetic material (incorporating BrdU), and divided, thereby completing the cell cycle. Thus, for this group, cell division occurred in a remarkably shorter period of time. A schematic representation is depicted in Figure 3(C).

### 3.5. *In vivo* studies exhibited divergent therapeutic profiles

**Figure 4.**
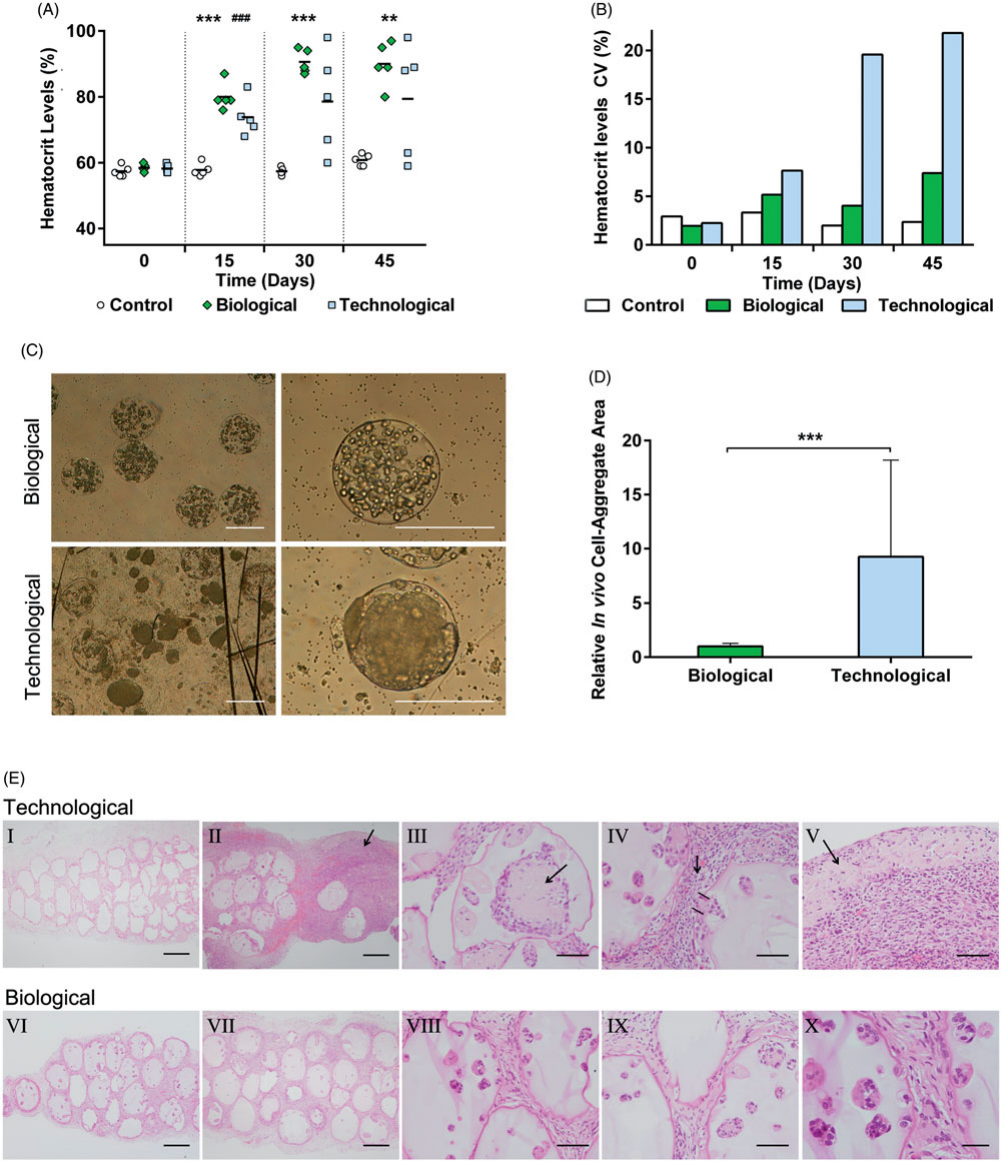
*In vivo* studies exhibited divergent therapeutic profiles. (A) Hematocrit levels increased progressively in a controlled fashion for all biological implants but showed an erratic trend with a big data dispersion for the technological group (B). (C) Morphological characterization after graft explantation revealed enormous cell-aggregates and broken capsules in the technological group. (D) Cell-aggregate area quantification proved statistically significant differences between the groups. (E) The histological studies showed empty microcapsules for the technological implants that failed (I). The analysis also confirmed that technological implants presented an intense inflammatory response (II), cell-aggregates causing microtumors (III), capsule rupture (IV), and the consequent extracapsular tumor formation (V). Such behavior was not observed in biological implants, where a lower inflammation (VI–VII) and capsule integrity with no cell extravasation (VIII–X) were observed. Graphs symbolize mean ± SD. (*n* = 5 for animal studies, *n* = 30 for diameter quantification). For the control group sole DPBS was administered. Statistical significance: (A) Between Control and Biological groups: **p < .01 and ***p < .001, between Control and Technological groups: ###*p* < .001; (D) ***p < .001. Scale bars: (C) = 400 µm; (E) I, II, VI, VII =400 µm; III, IV, V, VIII, IX =100 µm; X = 50 µm.

In order to assess the therapeutic effect *in vivo*, biological and technological microcapsules were implanted in C57BL/6 mice for 45 d. The biological group showed progressive increases in hematocrit levels, with narrowed distribution. Contrarily, technological microcapsules showed erratic profiles with high dispersion values (Figure 4(A,B)). Once the implants were retrieved, microscopic observation demonstrated that biological microcapsules maintained their spherical shape and integrity, but the vast majority of technological capsules were broken and released their load in form of enormous cell-aggregates (Figure 4(C)). Although cell-aggregates were also observed in the biological group, their size was significantly smaller (*p* < .001) (Figure 4(D)).

The hematoxylin and eosin staining revealed that all implants in the biological group showed a similar behavior; contrasting with technological implants, where the two grafts that failed to raise the hematocrit levels showed empty microcapsules (Figure 4(E.I)). The inflammatory response was remarkably more intense in the technological group (Figure 4(E.II)), except for the two implants showing capsules with no cell load (Figure 4(E.I)). Moreover, technological microcapsules showed enormous cell-aggregates, which caused mesenchymal micro tumors (Figure 4(E.III)). Broken capsules were observed in the technological group (Figure 4(E.IV)). This fact led to the release of the cellular content and subsequent inflammatory response. The rupture together with the aggressive proliferative capacity of this group caused cell migration and extracapsular tumor-like mass formation (Figure 4(E.V)). Such behavior was not observed in the biological group, were microcapsules maintained their integrity (Figure 4(E.VI–X).

### 3.6. Behavioral differences were caused at a genic level

For gene expression studies, four independent samples of cells within each type of matrix were analyzed. One technological replicate was removed from this expression analysis due to technical problems during the hybridization with the microarray. Our study recovered a set of 1570 genes with significant changes (*p* < .05) in expression. In particular, we focused on differentially expressed genes showing a FC >3 or < −3. Under such restriction, cells within technological microcapsules showed 176 up-regulated and 88 down-regulated genes versus the biological group (Supplementary Table 1). It is noteworthy that 86 out of the 264 sequences presented unknown function.

**Figure 5.**
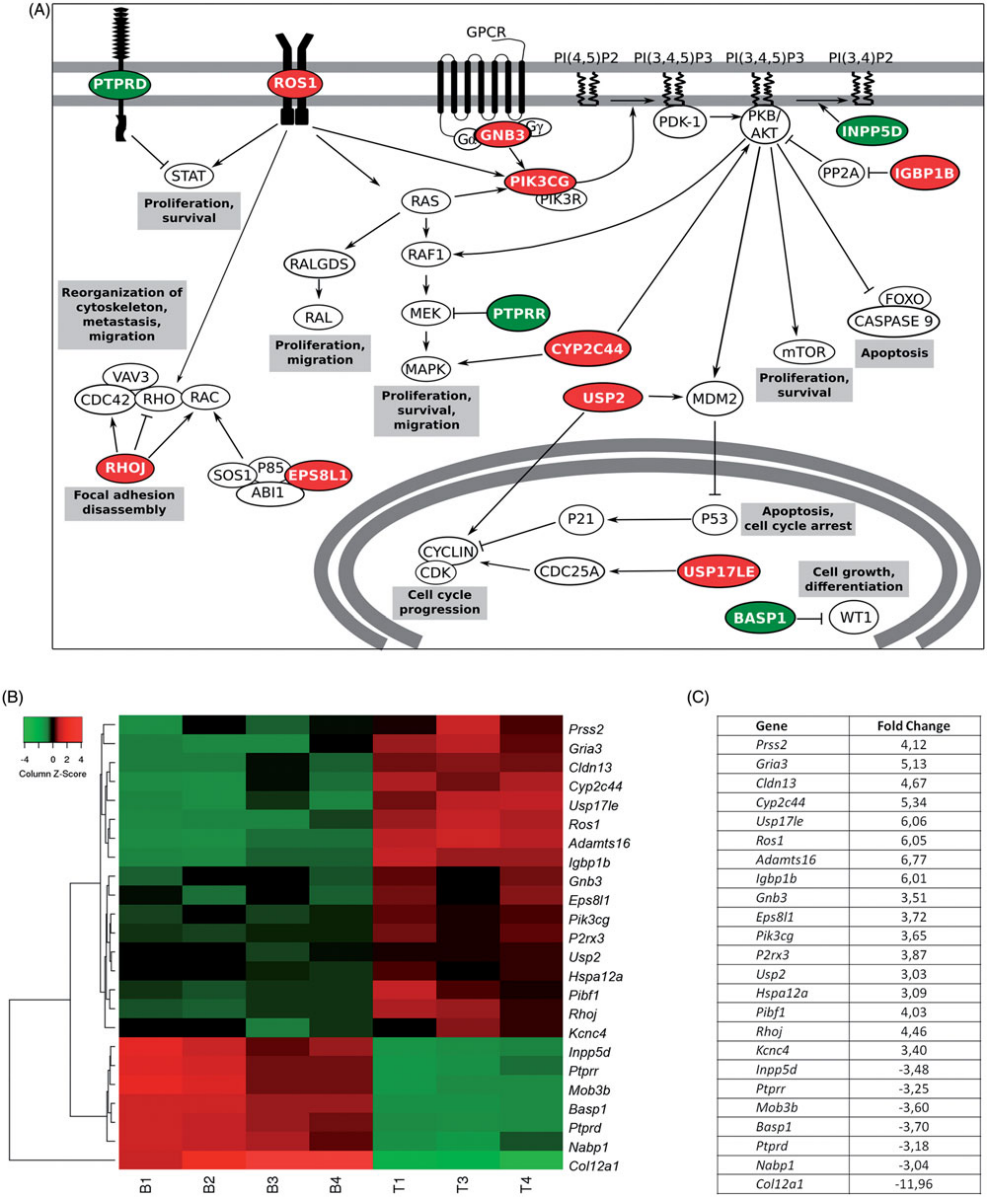
Gene expression studies proved that the behavioral differences were caused at a genic level. (A) Schematic representation of signaling pathways in which gene expression presents a fold change> 3 or< −3 in cells within technological microcapsules, in comparison to the biological group. (B) Heat map representation of differentially expressed genes. Results are expressed as a fold change ratio. Each column represents a replicate and each row represents a gene (Euclidean distance, average linkage; *n* = 4 for gene studies, with the loss of a technological replicate due to technical problems during hybridization process. B: biological; T: technological). (C) Values of fold change in cells within technological vs. biological capsules. In all cases, red color indicates up-regulated genes and green color down-regulated genes.

Among the rest, many of these genes pointed out to a higher activation of proliferative pathways in the technological group (Figure 5). Such is the case of the PI3K/Akt/mTOR pathway, where the up-regulation of genes such as *Gnb3* (FC 3.51), *Pik3cg* (FC 3.65), and *Igbp1b* (FC 6.01) together with the down-regulation of *Inpp5d* (FC −3.48) promoted this route. Similarly, the Ras/Raf/MAPK pathway was reinforced due to the up-regulation of *Ros1* (FC 6.05) or *Cyp2c44* (FC 5.34) genes. Additionally, the higher expression of *Usp2* (FC 3.03) and *Usp17le* (FC 6.06), together with the down-regulation of transcriptional co-repressors such as *Basp1* (FC −3.7) led to cell cycle progression.

The reorganization of the cytoskeleton was also enhanced in the technological group due to the up-regulation of genes such as *Rhoj* (FC 4.46) or *Eps8l1* (FC 3.72). Furthermore, the technological group showed up-regulation of the *Adamts16* gene (FC 6.77), suggesting chondrogenic differentiation. On the contrary, the biological group presented a high expression of *Col12a1* (FC −11.96), gene present in the osteogenic differentiation. Finally, the *Ptprd* (FC −3.18) and *Ptprr* (FC −3.25) tumor suppressor genes showed a lower expression in technological cells, whereas the *Hspa12a* heat shock protein was significantly up-regulated (FC 3.09).

## 4. Discussion

Currently, there is an urgent need to regulate cell behavior in encapsulation systems, since the lack of control leads to unsuccessful therapies and represents an important hurdle toward clinical translation (Bhujbal et al., 2014; Gonzalez-Pujana et al., 2017). Interestingly, the stiffness of the matrix has been described to play an important role in the regulation of cell responses (Shin & Mooney, 2016). Therefore, to date, many have been the attempts to modulate this factor. Nonetheless, little attention has been paid to the osmolarity adjusting agents as a tool to regulate the final outcome of the therapy. Considering that microcapsule formation occurs by electrostatic interactions, this process is directly influenced by the presence of electrolytes, especially divalent cations, in the surrounding media (Chen et al., 2014; Thu et al., 1996). Hence, we hypothesized that in the attempt to design solutions that meet the standards for cell culture, the choice of different types of osmolarity adjusting agents may play a pivotal role in the mechanics of the capsule. To shed light on this premise, two different sets of solutions were designed. Both of them were adjusted to adequate osmolarity values, however, different types of osmolarity adjusting agents were selected to do so. For the biological set of solutions, electrolytes such as sodium, potassium, calcium, or phosphates were chosen. Contrarily, mannitol was included as an inert agent in the technological set of solutions (Table 1). Subsequently, D1-MSCs-EPO was encapsulated using these sets, obtaining biological or technological microcapsules. This cell type was selected because of its aggressive proliferative capacity (Garate et al., 2015; Gurruchaga et al., 2015), which renders it a suitable model for studying the control that the mechanical configuration of the matrix may exert on cell behavior. Indeed, during the course of our study, these cells were able to proliferate even in starving conditions (Supplementary Figure S4).

Firstly, pivotal differences were observed when evaluating cell behavior *in vitro* (Figure 1). The morphological analysis, together with the BrdU uptake assay showed a significantly higher proliferation rate in the technological group, with the subsequent cell-aggregate formation (Figure 1(A–C)). These results could explain the so different tendencies that cells repeatedly followed in viability, metabolism and EPO secretion studies: linear for biological and exponential for technological microcapsules (Figure 1(D–F)). Moreover, regarding the technological group, the dramatic decrease of proliferation and the failure in the exponential trend by day 45 were probably caused by a decrease in viability (Figure 1(A,D)). We point out to the enormous aggregates as the most probable factor that originated cell death, possibly due to pore collapse (Leal-Egana et al., 2012), and subsequent limited diffusion of oxygen and nutrients to the inner core.

In order to determine if the contrasting cell behavior was originated by differences in the mechanical properties of the system, structural studies were carried out. Most importantly, these studies revealed significant differences in matrix configuration. Considering that the binding between the alginate and the crosslinking ion is responsible for conferring stiffness to the matrix, higher calcium levels, and thus a higher crosslinking degree, suggested a less permissive matrix in biological capsules (Figure 2(H)). According to these results, during the coating process, the calcium loss was lower when beads were put in contact with solutions that presented it, probably due to the gradient stabilization that could have prevented the leakage. Consequently, biological microcapsules retained more calcium, whereas the technological group presented a higher leakage of the ion, resulting in a more permissive matrix (Kleinberger et al., 2013; Ma et al., 1994). This property allowed an aggressive proliferation in the latter, leading to the formation of huge cell-aggregates. On the contrary, the more restrictive matrix of biological microcapsules established a control in cell division (Richardson et al., 2016; Liu et al., 2015). Therefore, it was not the direct effect of osmolarity adjusting agents on cells which evoked so contrasting behavior, but the effect this agents have in the capsule formation process and thus, in the final crosslinking degree of the alginate matrix.

Calcium determination may also explain the results obtained for particle size. When capsules were put in contact with technological solutions, the calcium loss not only led to a decreased stiffness, but also to swelling, thus increasing microcapsule diameter (Figure 2(A–C)) (Kleinberger et al., 2013). Additionally, a higher osmotic resistance in technological microcapsules suggested an enhanced interaction between the alginate matrix and the PLL coating (Figure 2(D–F)). This may have been caused by the contribution of two phenomena. Firstly, technological microcapsules presented less calcium in their matrix (Figure 2(H)), and consequently, a higher number of alginate chains were free to interact with the PLL. Secondly, since the membrane formation is governed by electrostatic interactions between the alginate and the polycation, the presence of ions in the surrounding media may weaken the bind (Thu et al., 1996). Therefore, although both groups presented a good osmotic resistance, it was enhanced in the technological group. Regarding permeability, no statistical differences were observed, with both groups maintaining an adequate MWCO to fulfill the objectives of the technology (Figure 2(G)).

To thoroughly evaluate the effect of the matrix crosslinking degree in cell division, next step was to study in depth cell cycle. The proliferation rate was remarkably enhanced for technological microcapsules by day 30 (Figure 3(A,B)). Although a 55.3% of biological and a 57.9% of technological cells presented a G0/1 state, interesting proliferative differences were detected in the remaining cells. Specifically, a significant number of cells in the biological group remained the 48 h after BrdU addition in the same S/G2/M state they were before inclusion of the thymidine analog. Contrarily, the technological group was able to duplicate the genetic material and complete the cycle, returning to G0/1 phase with the BrdU label incorporated in the DNA. This indicates that softer matrices present lower resistance to deformation, allowing a significantly faster cell division (Figure 3(C)) (Leal-Egana et al., 2012).

Because the *in vivo* studies represent the most similar approach to clinics, they were pivotal to determine if the obtained data remained significant. Concerning hematocrit profiles (Figure 4(A)), biological microcapsules showed a progressive increase, obtaining similar values for every mouse in the group. Nevertheless, by day 30, two of the technological implants had already failed, and the remaining showed important data dispersion (Figure 4(B)).

When morphologically analyzing the explants after 45 d of study, great differences were observed (Figure 4(C)). Important cell-aggregates were detected in technological microcapsules and their size quantification confirmed, once again, the divergent proliferation rates in each group (Figure 4(D)). Moreover, while biological microcapsules remained spherical and maintained their integrity, for the technological group, the majority of capsules were broken, allowing the cell content to be released to the surrounding tissue (Figure 4(C)). That was probably originated by the enormous aggregates whose aggressive growth triggered an increment in pressure that was not tolerated by the membranes of the system. Histological analysis supported these results by showing a significantly higher capsule rupture in the technological group, which resulted in tissue invasion and tumor-like mass formation (Figure 4(E.III–V)). This fact may have also contributed to the severe inflammatory response observed in such group (Figure 4(E.I–II)). In particular, dying cells released from technological microcapsules might have secreted DAMPs, which are extensively recognized to play a role in the responses against grafts (Paredes Juarez et al., 2014).

The final goal of cell microencapsulation technology is to maintain the bioactive factor levels within the therapeutic range in a sustained manner. Since biological implants provided progressive increases with minimal dispersion values, they were able to fulfill this objective and give rise to a controlled regimen. On the contrary, the erratic behavior shown by cells encapsulated within technological microcapsules led to an unpredictable secretion of EPO. Indeed, some implants failed and others maintained their functionality, making it difficult to foretell the result of the therapy. Moreover, it may be hypothesized that in the functional technological implants, due to the extreme proliferation rate allowed by such type of matrix, hematocrit levels may continue to increase, reaching toxic levels.

Microarray analysis explained that the contrasting cell behavior observed was due to differences at a genic level. Important proliferative pathways were significantly activated in cells enclosed within technological microcapsules. The up-regulation of genes such as *Gnb3*, *Pik3cg*, or *Igbp1b* together with the down-regulation of *Inpp5d*, enhanced the PI3K/Akt/mTor route. Similarly, the Ras/Raf/MAPK pathway was promoted due to over-expression of genes like *Ros1* (Chin et al., 2012) and *Cyp2c44*, which can also activate Akt (Yang et al., 2009). Interestingly, it has been reported that when these two pathways are mutated or amplified, proliferation, and survival signals are constitutively activated and, ultimately, lead to tumorigenesis (Wu et al., 2017). Additionally, the over-expression of *Ros1* might give rise to reorganization of the cytoskeleton, process often related to metastasis and migration (Chin et al., 2012). This effect may have been accentuated by the up-regulation of other implied genes such as *Rhoj*, which can evoke focal adhesion disassembly (Wilson et al., 2014), and *Eps8l1*, which can activate Rac, leading to the reorganization of the actin cytoskeleton (Offenhauser et al., 2004). Moreover, cell cycle arrest may have been repressed due to the up-regulation of *Usp2* and *Usp17le*, since they promote the stabilization of cyclin D1 (Shan et al., 2009) and Cdc25A (Hjortland & Mesecar, 2016), respectively. Supporting these results, *Ptprd* and *Ptprr* tumor suppressor genes were down-regulated in the technological group. The expression of the former can inhibit the Stat pathway, which leads to proliferation and survival (Ortiz et al., 2014). The latter, is able to inhibit MEK, repressing the Ras/Raf/MAPK route (Su et al., 2013). Furthermore, for the same group, we found up-regulation of *Hspa12a*, whose high expression has been proven in tumor tissues (Yang et al., 2015). Consequently, cells enclosed in technological microcapsules may have found less restriction to develop a tumor-like behavior.

Genes related to differentiation of D1-MSCs-EPO into other lineages presented a distinctive expression. In particular, we observed an up-regulation of *Col12a1* in biological microcapsules. This gene encodes type XII collagen, which is expressed by osteoblasts and localizes to areas of bone formation (Izu et al., 2011). On the other hand, the *Adamts16* gene, up-regulated in technological cells, has been reported to be expressed by MSCs during chondrogenesis (Boeuf et al., 2012). These differences may be explained by the already described mechanosensitive differentiation of MSCs by which, according to the physical properties of each matrix, cells differentiate into varying lineages (Rape et al., 2015).

Considering all these evidences, it is possible to determine that gene expression was considerably influenced by the mechanical characteristics of the matrix in which cells were encapsulated. The pivotal differences regarding expression resulted in a contrasting cell behavior both, *in vitro* and *in vivo*. Since in our system cells are responsible for producing the therapeutic factor, there is a direct connection between cell behavior and drug delivery. Therefore, the different cell responses had a drastic impact on the release of the therapeutic factor, influencing key points such as the efficacy and safety of the therapy. Therefore, our research supports the data described in the literature pointing out to a mechanosensing process in the absence of integrin binding domains (Huang et al., 2013; Bhujbal et al., 2014). Further research should focus on the mechanism by which it occurs in order to gain knowledge over the factors involved in cell-microenvironment interactions.

## 5. Conclusion

The present work provides new insights regarding the regulation of uncontrolled cell responses in alginate microspheres. In particular, we proved the employment of osmolarity adjusting agents as a useful tool to modify the mechanical configuration of the matrix, with no need of altering the biomaterial or cross linker type/proportion. Technological microcapsules, resulting from the employment of inert osmolarity adjusting agents, presented a permissive matrix that allowed uncontrolled cell division, with the subsequent erratic and dysfunctional therapeutic regimen. Contrarily, when employing electrolytes, including calcium or sodium, as osmolarity adjusting agents, biological capsules were formed. This type of matrix allowed establishing a tight control over cell proliferation, avoiding the enormous cell-aggregate formation, the risk of cell protrusion, the intense inflammatory response and the potential toxicity given by drug overdose. Overall, these data demonstrated that employing the biological formulation represents a valuable strategy to control cell behavior and thus achieve a predictable, safe and controlled *de novo* release of therapeutics.

## Supplementary Material

IDRD_Hernandez_et_al-Supplemental_Content.pdf
