# Supplementary material for: The role of osmolarity adjusting agents in the regulation of encapsulated cell behavior to provide a safer and more predictable delivery of therapeutics
Source: Drug Deliv. 2017 Oct 27;24(1):1654–66. doi: 10.1080/10717544.2017.1391894 (PMC8241175; doi:10.1080/10717544.2017.1391894)
Supplement: IDRD_Hernandez_et_al-Supplemental_Content.pdf [file IDRD_A_1391894_SM3028.pdf]

SUPPLEMENTARY DATA

Osmolarity adjusting agents play a key role in the regulation of encapsulated cell behavior providing a safer and more predictable delivery of therapeutics

Ainhoa Gonzalez-Pujana[a,b], Aitor Rementeria[c], Francisco Javier Blanco[d], Manoli Igartua[a,b], Jose Luis Pedraz[a,b], Edorta Santos-Vizcaino[a,b,*] and Rosa Maria Hernandez[a,b,*]

[a] NanoBioCel Group, Laboratory of Pharmaceutics, School of Pharmacy, University of the Basque Country (UPV/EHU).

[b] Biomedical Research Networking Centre in Bioengineering, Biomaterials and Nanomedicine (CIBER-BBN). Vitoria-Gasteiz, Spain.

[c] Department of Immunology, Microbiology and Parasitology, Faculty of Science and Technology, Fungal and Bacterial Biomics Research Group, University of the Basque Country (UPV/EHU), Leioa, Spain.

[d] CIBER-BBN-Bioscaff Cartílago, INIBIC Institute for Biomedical research, 15006, A Coruña, Spain.

*Corresponding authors at: Laboratory of Pharmaceutics, School of Pharmacy, University of the Basque Country (UPV/EHU); Paseo de la Universidad 7, 01006, Vitoria-Gasteiz, Spain. Fax number: +34 945013040.

R.M. Hernandez: rosa.hernandez@ehu.eus, +34 945013095.

E. Santos-Vizcaino: edorta.santos@ehu.eus, +34 945014518.

Supplementary Methods

Microarray hybridization and Transcriptomic analysis

Whole mouse gene expression microarray analysis was performed using the available SurePrint G3 Mouse GE v2 8x60K Microarray (Agilent Microarray Design ID: ID 074809, Agilent Technologies). Nucleic acid from each replicate, 100 ng, were labeled following the Agilent protocol "Gene Expression FFPE Workflow"**.** Briefly, cDNA library was generated and amplified using TransPlex Whole Transcriptome Amplification System (Sigma-Aldrich, Spain). The Titanium Taq DNA polymerase (Takara, Saint-Germain-en-Laye, France) was used during the cDNA library amplification step. Amplified cDNA was purified using the QIAquick PCR Purification kit (Qiagen, Hilden, Germany) and quantified using the NanoDrop 1000 spectrophotometer. The SureTag Labeling kit (Agilent Technologies) was used to enzymatically label 1.8 μg of amplified cDNA with cyanine 3-dUTP. Cy3-labeled cDNA samples were cleaned up using an Amicon Ultra-0.5 with Ultracel-30 Membrane, 30 kDa filter, provided with the SureTag Labeling kit. Yield and specific activity were determined using the NanoDrop 1000 spectrophotometer. The purified Cy3-labeled cDNA samples were hybridized to the Agilent SurePrint G3 Mouse GE v2 8x60K Microarray following the manufacturer instructions. Hybridized microarrays were scanned on a G2565CA DNA microarray scanner (Agilent Technologies).

Conventional statistical criteria (adjusted p-value < 0.05) were used for the selection of differentially expressed genes. For each differentially expressed gene the fold change (FC) was calculated as follows: $\log_2$ (FC) = $\log_2$ (Technological data) – $\log_2$ (Biological data), and the absolute FC was calculated according to the following formula [1]: if $\log_2$ (FC) > 0, FC = $2^{(\log2(FC))}$; if $\log_2$ (FC) < 0, FC = (-1) x $2^{-(\log2(FC))}$. Positive and negative values of FC showed up-regulated or down-regulated genes on Technological microcapsules, respectively.

All microarray procedures were performed at the Gene Expression Unit of the Genomics Facility in the UPV/ EHU (SGIker platforms; Leioa, Spain). PANTHER Gene List Analysis Tools (http://pantherdb.org/) and National Center for Biotechnology Information databases (https://www.ncbi.nlm.nih.gov/) were used to detect the biological activity of genes. The heat maps and clustering of differentially expressed genes were constructed using Heatmapper (www.heatmapper.ca) [2]. For gene and protein nomenclature, the guidelines of Mouse Nomenclature Home Page of Mouse Genome Informatics (MGI) were followed (http://informatics.jax.org/mgihome/nomen/).

References

[1] N. Leonhardt, J.M. Kwak, N. Robert, D. Waner, G. Leonhardt, J.I. Schroeder, Microarray expression analyses of Arabidopsis guard cells and isolation of a recessive abscisic acid hypersensitive protein phosphatase 2C mutant, Plant Cell. 16 (2004) 596-615.

[2] S. Babicki, D. Arndt, A. Marcu, Y. Liang, J.R. Grant, A. Maciejewski, D.S. Wishart, Heatmapper: web-enabled heat mapping for all, Nucleic Acids Res. 44 (2016) W147-53.

Supplementary figures

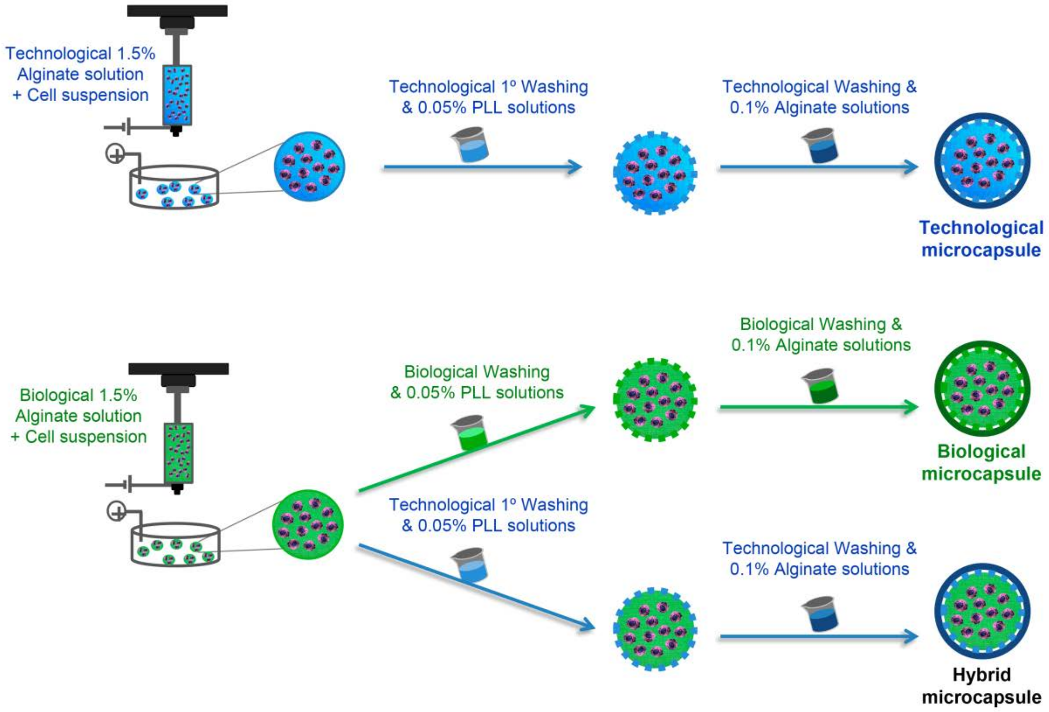

Supplementary S1. ***Schematic representation of the synthesis of Biological, Technological and Hybrid microcapsules.*** PLL - Poly-L-Lysine

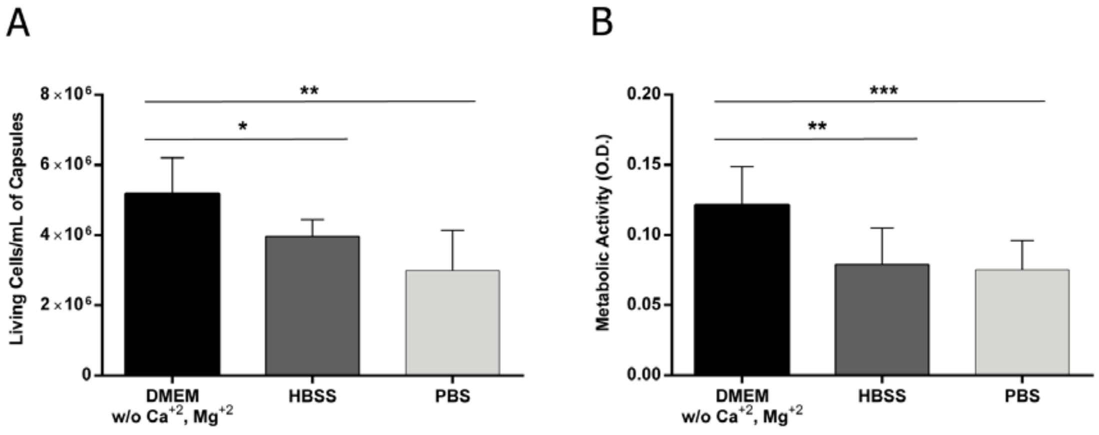

Supplementary S2. ***Preliminary studies to design the Biological 1.5% Alginate solution*****.** Live/dead flow cytometry and CCK-8 metabolic assays were performed to select the most suitable vehicle containing electrolytes for the Biological 1.5% Alginate solution. Among them, DMEM w/o Ca^{2+}, Mg^{+2} demonstrated to provide the best results for cell viability and metabolic activity. Graphs symbolize mean ± SD (n = 3 for cell viability studies, n = 7 for metabolic assays). Statistical significance: *$p < 0.05$, **$p < 0.01$ and ***$p < 0.001$. DMEM - Dulbecco's modified Eagle's medium; HBSS - Hanks balanced salt solution; PBS - Phosphate buffered saline.

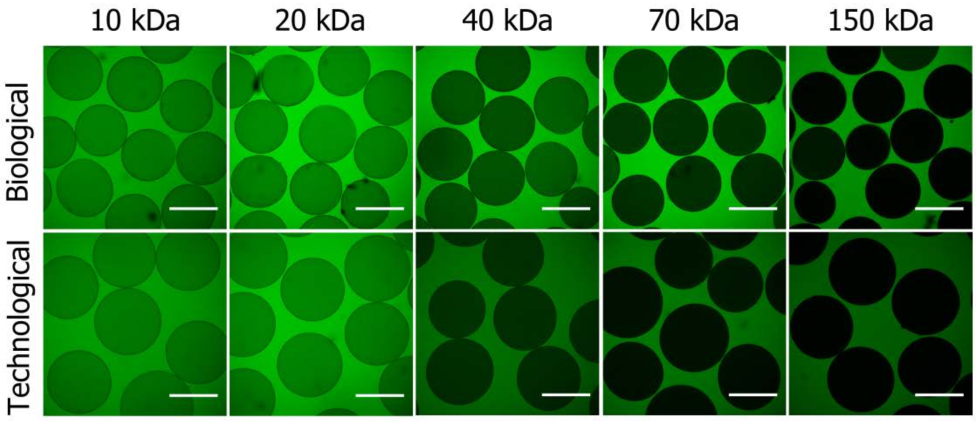

Supplementary S3. ***Microcapsule permeability.*** FITC-labeled dextran diffusion (n = 4) showed no statistically significant differences in the molecular weight cut off. Scale bars = 400 µm.

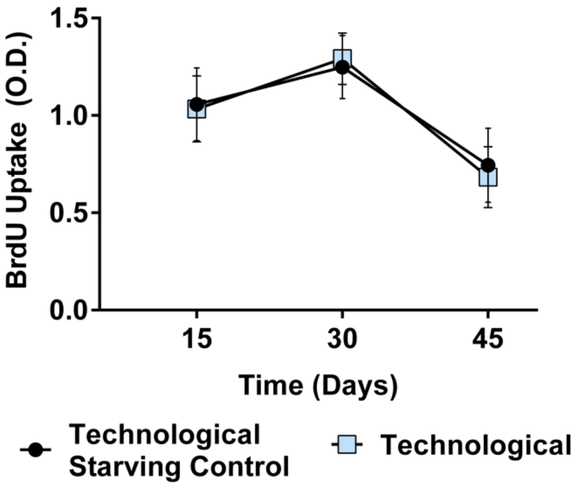

Supplementary S4. ***D1-MSCs present an aggressive proliferative capacity.*** During bromodeoxyuridine (BrdU) uptake assays, when comparing results of Technological samples (incubated in DMEM with 10% FBS supplementation) to the starving control (same Technological microcapsules incubated in DMEM with 0.1% FBS supplementation) no statistical differences were found, confirming the high proliferative capacity of these cells. Graphs symbolize mean ± SD (n = 5). DMEM - Dulbecco's modified Eagle's medium, FBS - fetal bovine serum.

Supplementary Table 1. ***Sequences that showed significant expression differences (p < 0.05) and a Fold Change > 3 or < -3 in the analysis of microarray data.***

**Probe Name[a]**	**Gene Symbol**	**Gene Name**	**Entrez Gene ID[b]**	**UniGene ID[c]**	**Gen Ontology (GO) code[d]**	**Log_2 Technological samples (mean ± std.dev)**	**Log_2 Biological samples (mean ± std.dev)**	**Fold Change[e]**
A_30_P01026742						1.94 ± 0.39	-1.45 ± 0.84	10.50
A_52_P395083				Mm.386943		1.77 ± 0.83	-1.33 ± 1.08	8.60
A_51_P263719	*Vmn1r193*	Vomeronasal 1 receptor 193	171259	Mm.432535	GO:0005550, GO:0016503, GO:0007186, GO:0005887, GO:0019236	1.70 ± 0.64	-1.28 ± 1.05	7.87
A_51_P379807	*Cyp4f41-ps*	Cytochrome P450, family 4, subfamily f, polypeptide 41 pseudogene	77875	Mm.245027		1.70 ± 0.64	-1.27 ± 0.09	7.84
A_55_P2041444	*Xlr3a*	X-linked lymphocyte-regulated 3A	22445	Mm.475098	GO:0008150, GO:0003674, GO:0007286, GO:0005634, GO:0005575	1.70 ± 0.54	-1.27 ± 0.11	7.82
A_51_P402160	*Zfp750*	Zinc finger protein 750	319530	Mm.11395	GO:0006355, GO:0001077, GO:0008544, GO:0006366, GO:0045944, GO:0005730, GO:0005634, GO:0001046, GO:0046872, GO:0030154, GO:0006351, GO:0043231	1.67 ± 0.30	-1.26 ± 1.24	7.63
A_55_P2081966	*Slc22a20*	Solute carrier family 22 (organic anion transporter), member 20	381203	Mm.329638	GO:0016020, GO:0022857, GO:0015711, GO:0006810, GO:0008514, GO:0016021, GO:0055085, GO:0006811	1.66 ± 0.37	-1.24 ± 0.31	7.46
A_55_P2804426	*Pid1*	Phosphotyrosine interaction domain containing 1	98496	Mm.187470	GO:2001170, GO:0005515, GO:0090298, GO:2001171, GO:0046627, GO:0051881, GO:0070346, GO:2000045, GO:2001274, GO:0071398, GO:0010628, GO:0005737, GO:0070584, GO:0071345, GO:0045944, GO:0071356, GO:0044320, GO:0046325, GO:0090005, GO:0071354, GO:0001933, GO:0005654, GO:2000377, GO:2000379	1.63 ± 1.54	-1.22 ± 0.47	7.19
A_55_P2526140	*Adamts16*	A disintegrin-like and metallopeptidase (reprolysin type) with thrombospondin type 1 motif, 16	271127		GO:0001658, GO:0005578, GO:0005576, GO:0005575, GO:0046872, GO:0003073, GO:0031012, GO:0003674, GO:0016787, GO:0006508, GO:0008233, GO:0008270, GO:0004222, GO:0008237	1.58 ± 0.42	-1.18 ± 0.11	6.77
A_52_P456458				Mm.441109		1.57 ± 0.52	-1.17 ± 0.11	6.69
A_52_P570679	*Scgb2b24*	Secretoglobin, family 2B, member 24	233090	Mm.261648	GO:0008150, GO:0003674, GO:0005576, GO:0005575, GO:0005615	1.55 ± 1.02	-1.16 ± 0.11	6.54
A_30_P01026842						1.50 ± 0.77	-1.12 ± 1.11	6.17
A_51_P244020	*Rtbdn*	Retbindin	234542	Mm.461701	GO:0008150, GO:0003674, GO:0005576, GO:0005575	1.49 ± 0.71	-1.12 ± 0.19	6.11
A_51_P372253	*Ackr2*	Atypical chemokine receptor 2	59289	Mm.258105	GO:0005515, GO:0070098, GO:0005886, GO:0004930, GO:0005884, GO:0019957, GO:0006898, GO:0007165, GO:0006954, GO:0016020, GO:0006935, GO:0007186, GO:0016494, GO:0004950, GO:0016493, GO:0042119, GO:0016021, GO:0004871, GO:0005044, GO:0005768	1.49 ± 0.82	-1.11 ± 0.89	6.07
A_52_P345966	*Usp17le*	Ubiquitin specific peptidase 17-like E	625530	Mm.14130	GO:0004197, GO:0016579, GO:0004843, GO:0061136, GO:0043161, GO:0005634	1.49 ± 0.56	-1.11 ± 0.48	6.06
A_51_P453376	*Ros1*	Ros1 proto-oncogene	19886	Mm.236163	GO:0032006, GO:0048471, GO:0005886, GO:0008283, GO:0007283, GO:0030154, GO:0002066, GO:0000166, GO:0016301, GO:0070372, GO:0038083, GO:0010966, GO:0016772, GO:0023014, GO:0009986, GO:0019903, GO:0018108, GO:0005524, GO:0016310, GO:0004672, GO:0016020, GO:0010629, GO:0016049, GO:0006468, GO:0004713, GO:0016021, GO:0004714, GO:0016740	1.48 ± 0.48	-1.11 ± 0.16	6.05
A_52_P413134	*Igbp1b*	Immunoglobulin (CD79A) binding protein 1b	50540	Mm.84043	GO:0005515, GO:0042113, GO:0007165, GO:0050790, GO:0005737, GO:0019208, GO:0009966, GO:0005575	1.48 ± 0.11	-1.11 ± 0.13	6.01
A_51_P101347	*Pls1*	Plastin 1 (I-isoform)	102502	Mm.11869	GO:0005515, GO:0032532, GO:0030033, GO:0005509, GO:0003779, GO:0046872, GO:0005903, GO:0005737, GO:0051015, GO:0001951, GO:0040018, GO:0070062, GO:0090004	1.42 ± 0.77	-1.07 ± 0.74	5.61
A_52_P490964				Mm.417805		1.41 ± 0.18	-1.06 ± 0.41	5.52
A_55_P2041559	*Olfr655*	Olfactory receptor 655	258817	Mm.377706	GO:0050911, GO:0007608, GO:0004984, GO:0007186, GO:0016021	1.39 ± 0.98	-1.05 ± 0.67	5.42
A_52_P240632	*Olfr633*	Olfactory receptor 633	258351	Mm.377448	GO:0050911, GO:0007608, GO:0004984, GO:0007186, GO:0016021	1.38 ± 0.62	-1.04 ± 0.39	5.36
A_55_P2108526	*LOC102639117*	Secretoglobin family 2A member 2-like	102639117			1.38 ± 0.31	-1.04 ± 0.14	5.34
A_66_P104979	*Cyp2c44*	Cytochrome P450, family 2, subfamily c, polypeptide 44	226143	Mm.329866	GO:0008392, GO:0005737, GO:0006805, GO:0019825, GO:0042738, GO:0019373, GO:0008395, GO:0016712, GO:0055114, GO:0043231, GO:0020037	1.38 ± 0.16	-1.04 ± 0.78	5.34
A_52_P1067724				Mm.403098		1.38 ± 0.54	-1.03 ± 0.45	5.32
A_51_P199475	*Siglecf*	Sialic acid binding Ig-like lectin F	233186	Mm.290569	GO:0016020, GO:0030246, GO:0016021, GO:0048029, GO:0007155	1.37 ± 0.30	-1.03 ± 0.12	5.26
A_55_P2085989	*Gm13119*	Predicted gene 13119	433779	Mm.389596	GO:0008150, GO:0003674, GO:0005575	1.35 ± 0.43	-1.01 ± 0.11	5.13

A_66_P132292	*Gria3*	Glutamate receptor, ionotropic, AMPA3 (alpha 3)	53623	Mm.327681	GO:0005515, GO:0030054, GO:0005886, GO:0005234, GO:0045202, GO:0030425, GO:0032279, GO:0034220, GO:0043197, GO:0043198, GO:0045211, GO:0004971, GO:0043195, GO:0004970, GO:0032281, GO:0006810, GO:0043025, GO:0043204, GO:0004872, GO:0006811, GO:0035249, GO:0014069, GO:0043083, GO:0030165, GO:0043234, GO:0035235, GO:0001919, GO:0016020, GO:0016021, GO:0005216	1.35 ± 0.49	-1.01 ± 0.73	5.13
A_66_P124942	*Gm35705*	Predicted gene, 35705	102639378	Mm.55702		1.34 ± 0.65	-1.00 ± 0.10	5.06
A_66_P111435	*Faddos*	Fas (TNFRSF6)-associated via death domain, opposite strand	100038412	Mm.413024		1.32 ± 0.10	-0.99 ± 0.64	4.99
A_55_P2737124	*Ryr2*	Ryanodine receptor 2, cardiac	20191	Mm.239871	GO:0060401, GO:0005515, GO:0005516, GO:0005513, GO:0030018, GO:0060402, GO:0035584, GO:0019722, GO:0043621, GO:0060048, GO:0030017, GO:0042802, GO:0005622, GO:0003143, GO:0034220, GO:0006810, GO:0032026, GO:0015278, GO:0070062, GO:0005262, GO:0051209, GO:0019901, GO:0014850, GO:0005509, GO:0071313, GO:0035994, GO:0007275, GO:0014808, GO:0030509, GO:0043924, GO:0005790, GO:0031234, GO:0051592, GO:0043234, GO:0002027, GO:0070588, GO:0055117, GO:0010460, GO:0019899, GO:0005216, GO:0031672, GO:0071421, GO:0005219, GO:0005218, GO:0034236, GO:0043005, GO:0034237, GO:0005886, GO:0005783, GO:0070296, GO:0005635, GO:0005829, GO:0051284, GO:0003300, GO:0003220, GO:0005737, GO:0031000, GO:0006816, GO:0060070, GO:0006811, GO:0001666, GO:0048763, GO:0051480, GO:0051481, GO:0006874, GO:0005623, GO:0010882, GO:0010881, GO:0033017, GO:0034704, GO:0055085, GO:0042383, GO:0016529, GO:0016020, GO:0051775, GO:0016021	1.31 ± 0.32	-0.98 ± 0.12	4.91
A_55_P2185628				Mm.149465		1.31 ± 0.77	-0.98 ± 0.81	4.91
A_55_P2725464						1.31 ± 0.37	-0.98 ± 0.24	4.90
A_52_P401535	*Cd209d*	CD209d antigen	170779	Mm.111026	GO:0016020, GO:0030246, GO:0050715, GO:0016021, GO:0005537, GO:0046872, GO:0042803, GO:0042742, GO:0006897	1.30 ± 0.60	-0.98 ± 0.04	4.86
A_52_P525989	*4930584F24Rik*	RIKEN cDNA 4930584F24 gene	75942	Mm.50087	GO:0008150, GO:0003674, GO:0005575	1.29 ± 0.16	-0.97 ± 0.22	4.77
A_66_P114678	*Gm9915*	Predicted gene 9915	791306	Mm.491571		1.28 ± 0.03	-0.96 ± 0.67	4.72
A_51_P469568	*Cldn13*	Claudin 13	57255	Mm.86652	GO:0030054, GO:0016020, GO:0005886, GO:0005198, GO:0016021, GO:0005923, GO:0016328	1.27 ± 0.31	-0.95 ± 0.67	4.67
A_55_P2345726	*D030025E07Rik*	RIKEN cDNA D030025E07 gene	402774	Mm.209170		1.26 ± 0.51	-0.95 ± 0.39	4.63
A_51_P502521	*9530002B09Rik*	RIKEN cDNA 9530002B09 gene	77432	Mm.491436	GO:0002084, GO:0008474, GO:0005764, GO:0006898, GO:0007399	1.26 ± 0.52	-0.94 ± 0.60	4.60
A_55_P2925449	*Ccser1*	Coiled-coil serine rich 1	232035	Mm.487153	GO:0008150, GO:0003674, GO:0005575	1.25 ± 0.93	-0.94 ± 0.21	4.54
A_52_P191633	*Fam71b*	Family with sequence similarity 71, member B	432552	Mm.443244	GO:0008150, GO:0003674, GO:0005634	1.25 ± 0.55	-0.93 ± 0.10	4.54
A_52_P149173	*Vmn2r53*	Vomeronasal 2, receptor 53	637908	Mm.469871		1.24 ± 0.64	-0.93 ± 0.71	4.51
A_55_P2317435	*AI594674*	Expressed sequence AI594674	98401	Mm.441861		1.24 ± 0.93	-0.93 ± 0.43	4.51
A_55_P2176076	*Lrrc7*	Leucine rich repeat containing 7	242274	Mm.132162	GO:0043005, GO:0030054, GO:0005886, GO:0014069, GO:0045202, GO:0030175, GO:0008022, GO:0015629, GO:0043194, GO:0005737, GO:0016020, GO:0043197, GO:0045211, GO:0010976	1.24 ± 0.38	-0.93 ± 0.52	4.48
A_66_P134649	*Olfr798*	Olfactory receptor 798	258549	Mm.463730	GO:0050911, GO:0007608, GO:0004984, GO:0007186, GO:0016021	1.23 ± 0.21	-0.93 ± 0.10	4.47
A_55_P2000196	*Rhoj*	Ras homolog gene family, member J	80837	Mm.27467	GO:0005515, GO:0030036, GO:0005886, GO:0007264, GO:0008360, GO:0003924, GO:0061299, GO:0007266, GO:0005525, GO:0005622, GO:0000166, GO:0016020, GO:0070062	1.23 ± 0.60	-0.92 ± 0.13	4.46
A_52_P590995	*E230016K23Rik*	RIKEN cDNA E230016K23 gene	100504464	Mm.341706	GO:0008150, GO:0003674, GO:0005575	1.23 ± 0.63	-0.92 ± 0.11	4.44
A_66_P103233				Mm.406426		1.21 ± 0.46	-0.91 ± 0.87	4.35
A_55_P2083694	*Adam33*	A disintegrin and metallopeptidase domain 33	110751	Mm.108550	GO:0042035, GO:0016787, GO:0016020, GO:0042127, GO:0006508, GO:0008270, GO:0008233, GO:0016021, GO:0004222, GO:0046872, GO:0008237	1.21 ± 0.96	-0.91 ± 0.12	4.33
A_30_P01018196						1.20 ± 0.28	-0.90 ± 0.09	4.30
A_52_P598862	*Gm23952*	Predicted gene, 23952	102634376	Mm.259768		1.20 ± 0.18	-0.90 ± 0.79	4.28
A_55_P2588947	*Spink10*	Serine peptidase inhibitor, Kazal type 10	328971	Mm.312675	GO:0008150, GO:0003674, GO:0016020, GO:0004867, GO:0010466, GO:0016021, GO:0005575, GO:0030414	1.19 ± 0.77	-0.90 ± 0.82	4.26
A_51_P482322	*Vmn1r210*	Vomeronasal 1 receptor 210	171269	Mm.377221	GO:0005550, GO:0016503, GO:0007186, GO:0005887, GO:0019236	1.19 ± 0.88	-0.90 ± 0.11	4.25
A_65_P13823	*Myoz2*	Myozenin 2	59006	Mm.141157	GO:0030018, GO:0005737, GO:0031433, GO:0003779, GO:0015629	1.18 ± 0.59	-0.89 ± 0.08	4.20
A_55_P2150876	*Als2cr12*	Amyotrophic lateral sclerosis 2 (juvenile) chromosome region, candidate 12 (human)	108812	Mm.442063	GO:0008150, GO:0003674, GO:0005737, GO:0035686, GO:0036126, GO:0001520	1.18 ± 0.31	-0.88 ± 0.07	4.18

A_55_P1999483	*Dsc3*	Desmocollin 3	13507	Mm.89935	GO:0030057, GO:0030054, GO:0005886, GO:0045295, GO:0005509, GO:0050821, GO:0005576, GO:0001701, GO:0046872, GO:0005737, GO:0016020, GO:0016021, GO:0007155, GO:0007156	1.18 ± 0.38	-0.88 ± 0.08	4.17
A_66_P109431	*Tmem125*	Transmembrane protein 125	230678	Mm.23639	GO:0008150, GO:0003674, GO:0016020, GO:0016021, GO:0005575	1.18 ± 0.52	-0.88 ± 0.18	4.17
A_55_P2345314	*1700003P14Rik*	RIKEN cDNA 1700003P14 gene	75890	Mm.141235		1.17 ± 0.25	-0.88 ± 0.13	4.14
A_51_P224868	*4930456L15Rik*	RIKEN cDNA 4930456L15 gene	71650	Mm.158744		1.17 ± 0.99	-0.88 ± 0.83	4.14
A_55_P2386236	*Ceacam2*	Carcinoembryonic antigen-related cell adhesion molecule 2	26367	Mm.439731	GO:0005886, GO:0009986, GO:0019901, GO:2000252, GO:0042803, GO:0070348, GO:0016020, GO:0016032, GO:0016021, GO:0007156, GO:2000505, GO:0009897, GO:0070062	1.17 ± 0.31	-0.88 ± 0.75	4.14
A_52_P427377	*Sez6l*	Seizure related 6 homolog like	56747	Mm.143742	GO:0008344, GO:0003674, GO:0016020, GO:0005886, GO:0090036, GO:0021680, GO:0005783, GO:0043025, GO:0016021, GO:0060074	1.17 ± 0.86	-0.88 ± 0.16	4.13
A_51_P418901	*Prss2*	Protease, serine 2	22072	Mm.276926	GO:0004252, GO:0005509, GO:0007586, GO:0005576, GO:0005615, GO:0046872, GO:0031000, GO:0016787, GO:0030574, GO:0006508, GO:0008236, GO:0008233, GO:0035094	1.17 ± 1.02	-0.88 ± 0.74	4.12
A_55_P2062589	*Mtnr1a*	Melatonin receptor 1A	17773	Mm.5133	GO:0005886, GO:0004930, GO:0042562, GO:0043235, GO:0007165, GO:0007186, GO:0016020, GO:0005887, GO:0043025, GO:0007623, GO:0097159, GO:0030828, GO:0004871, GO:0008502, GO:0016021, GO:0046676	1.17 ± 0.57	-0.87 ± 0.15	4.11
A_55_P2278775	*A1cf*	APOBEC1 complementation factor	69865	Mm.5307	GO:0030895, GO:0005515, GO:0006397, GO:0003727, GO:0016556, GO:0005783, GO:0045293, GO:0003723, GO:0016554, GO:0050821, GO:0005634, GO:0003676, GO:0003729, GO:0010609, GO:0005720, GO:0005737, GO:0000166, GO:0005654	1.16 ± 0.23	-0.87 ± 0.06	4.10
A_66_P133740	*2810433D01Rik*	RIKEN cDNA 2810433D01 gene	77132	Mm.194729		1.16 ± 0.29	-0.87 ± 0.61	4.09
A_65_P12528	*Oas1c*	2'-5' oligoadenylate synthetase 1C	114643	Mm.43230	GO:0003725, GO:0008150, GO:0001730, GO:0005575	1.16 ± 0.16	-0.87 ± 0.70	4.09
A_30_P01031873						1.16 ± 0.50	-0.87 ± 0.36	4.06
A_52_P593015	*Pibf1*	Progesterone immunomodulatory binding factor 1	52023	Mm.22835	GO:0008150, GO:0003674, GO:0005813	1.15 ± 0.71	-0.86 ± 0.12	4.03
A_55_P2403144	*4930548J01Rik*	RIKEN cDNA 4930548J01 gene	78043	Mm.398313		1.14 ± 0.42	-0.86 ± 0.19	4.00
A_55_P2789059	*1700096J18Rik*	RIKEN cDNA 1700096J18 gene	67927	Mm.394490		1.14 ± 0.35	-0.85 ± 0.44	3.97
A_51_P451696						1.12 ± 0.64	-0.84 ± 0.33	3.88
A_51_P240723	*1700022A21Rik*	Glycerol-3-phosphate dehydrogenase 1-like pseudogene	72252	Mm.87446		1.12 ± 0.77	-0.84 ± 0.18	3.88
A_55_P2375194	*P2rx3*	Purinergic receptor P2X, ligand-gated ion channel, 3	228139	Mm.448935	GO:0043005, GO:0030424, GO:0009408, GO:0009409, GO:0007268, GO:0019228, GO:0005639, GO:0009266, GO:0009743, GO:0050909, GO:0043197, GO:0048167, GO:0043195, GO:0043025, GO:0006810, GO:0033198, GO:0001614, GO:0051260, GO:0014832, GO:0006812, GO:0006811, GO:0035590, GO:0005791, GO:0001666, GO:0005794, GO:0010033, GO:0004931, GO:0007274, GO:0030432, GO:0005524, GO:0009612, GO:0043235, GO:0016020, GO:0005887, GO:0045121, GO:0016021, GO:0048266, GO:0005216	1.12 ± 0.06	-0.84 ± 0.14	3.87
A_55_P1959496	*Sbno2*	Strawberry notch homolog 2 (Drosophila)	216161	Mm.262102	GO:0005515, GO:0006355, GO:0072674, GO:0072675, GO:0061430, GO:0030316, GO:0050727, GO:0005575, GO:0030282, GO:0006351, GO:0002281, GO:0045944, GO:0045892	1.11 ± 0.82	-0.83 ± 0.38	3.86
A_55_P1954226	*Olfr1018*	Olfactory receptor 1018	258579	Mm.223106	GO:0050911, GO:0007608, GO:0004984, GO:0007186, GO:0016021	1.11 ± 0.30	-0.83 ± 0.07	3.86
A_55_P2018697	*Ldhd*	Lactate dehydrogenase D	52815	Mm.27589	GO:0005739, GO:0005515, GO:0004458, GO:0006754, GO:0008762, GO:0050660, GO:0016491, GO:0008720, GO:0005743, GO:0016614, GO:0003824, GO:0055114	1.11 ± 0.06	-0.83 ± 0.77	3.85
A_52_P108401	*Tspear*	Thrombospondin type laminin G domain and EAR repeats	252974	Mm.221237	GO:0003674, GO:0060170, GO:0009986, GO:0007605, GO:0032420, GO:0005576, GO:0042995	1.11 ± 0.61	-0.83 ± 0.48	3.84
A_52_P605596	*Olfr107*	Olfactory receptor 107	258504	Mm.377541	GO:0050911, GO:0007608, GO:0050907, GO:0004984, GO:0007186, GO:0016021, GO:0004888	1.11 ± 0.36	-0.83 ± 0.13	3.84
A_55_P2727223				Mm.211290		1.10 ± 0.56	-0.83 ± 0.16	3.80
A_52_P246992	*4930558C23Rik*	RIKEN cDNA 4930558C23 gene	67654	Mm.75066		1.10 ± 0.36	-0.82 ± 0.12	3.78
A_66_P112448				Mm.321371		1.09 ± 0.15	-0.82 ± 0.51	3.77
A_55_P2551230	*Gm29571*	predicted gene 29571	102640597	Mm.412041		1.09 ± 0.47	-0.82 ± 0.11	3.77
A_66_P137376						1.09 ± 0.24	-0.82 ± 0.11	3.76
A_55_P2153753	*5830411N06Rik*	RIKEN cDNA 5830411N06 gene	244234	Mm.85125	GO:0003674, GO:0032620, GO:0002365, GO:0009897	1.08 ± 0.86	-0.81 ± 0.34	3.73
A_52_P238468	*Gipr*	Gastric inhibitory polypeptide receptor	381853	Mm.333633	GO:0005886, GO:0004930, GO:0030819, GO:0007204, GO:0005829, GO:0051592, GO:0007165, GO:0016519, GO:0007186, GO:0016020, GO:0007218, GO:0031018, GO:0002029, GO:0032024, GO:0004872, GO:0016021, GO:0004871, GO:0017046, GO:0007166, GO:0004888	1.08 ± 0.29	-0.81 ± 0.63	3.73

A_52_P37757	*Eps8l1*	EPS8-like 1	67425	Mm.360392	GO:0035023, GO:0003779, GO:0032587, GO:0007266, GO:0001726, GO:0005622, GO:0043547, GO:0043234, GO:0005737, GO:0016601, GO:0042608, GO:1900029, GO:0051015, GO:0005089, GO:0030676, GO:0070062	1.08 ± 0.33	-0.81 ± 0.76	3.72
A_55_P2064731	*Npbwr1*	Neuropeptides B/W receptor 1	226304	Mm.291110	GO:0043005, GO:0005886, GO:0004930, GO:0019222, GO:0007268, GO:0008188, GO:0007165, GO:0042923, GO:0007186, GO:0016020, GO:0005887, GO:0007187, GO:0007218, GO:0004871, GO:0016021	1.08 ± 0.15	-0.81 ± 0.55	3.72
A_52_P292404	*Apoa5*	Apolipoprotein A-V	66113	Mm.29738	GO:0006695, GO:0050996, GO:0031102, GO:0017127, GO:0042627, GO:0010873, GO:0070328, GO:0010898, GO:0072562, GO:0010902, GO:0030300, GO:0070325, GO:0005615, GO:0060229, GO:0060228, GO:0008289, GO:0034361, GO:0005543, GO:0015485, GO:0034364, GO:0034380, GO:0006810, GO:0019433, GO:0008201, GO:0042632, GO:0033344, GO:0045723, GO:0043691, GO:0031210, GO:0005576, GO:0060230, GO:0034370, GO:0033700, GO:0034372, GO:0006869, GO:0042157, GO:0006641, GO:0055090, GO:0046470, GO:0035473, GO:0008047, GO:0019899, GO:0051006, GO:0050750	1.08 ± 0.15	-0.81 ± 0.15	3.72
A_66_P130576	*LOC73899*	Uncharacterized LOC73899	73899	Mm.393239		1.08 ± 0.45	-0.81 ± 0.39	3.71
A_51_P314107	*Gsdma*	Gasdermin A	57911	Mm.458085	GO:0003674, GO:0048471, GO:0005737, GO:0006915	1.08 ± 0.45	-0.81 ± 0.17	3.69
A_55_P2057761	*Olfr971*	Olfactory receptor 971	258607	Mm.377589	GO:0050911, GO:0007608, GO:0004984, GO:0007186, GO:0005887, GO:0004930, GO:0005549, GO:0016021	1.07 ± 0.30	-0.81 ± 0.10	3.68
A_30_P01017727						1.07 ± 0.68	-0.80 ± 0.23	3.68
A_51_P507832	*Pik3cg*	Phosphoinositide-3-kinase, catalytic, gamma polypeptide	30955	Mm.101369	GO:0051897, GO:0005886, GO:2000270, GO:0097284, GO:0010897, GO:0007204, GO:0010818, GO:0005829, GO:0005737, GO:0006954, GO:0002675, GO:0000166, GO:0006935, GO:0043406, GO:0016301, GO:0016303, GO:0016773, GO:0016772, GO:0036092, GO:0048015, GO:0030593, GO:0030168, GO:0032252, GO:0001525, GO:0043085, GO:0005524, GO:0014065, GO:0006897, GO:0070527, GO:0002250, GO:0046854, GO:0016310, GO:0046875, GO:0007186, GO:0016020, GO:0046934, GO:0004674, GO:0045087, GO:0035005, GO:0001932, GO:0006468, GO:0071320, GO:0005942, GO:0016740, GO:0005944, GO:0002376	1.07 ± 0.03	-0.80 ± 0.39	3.65
A_52_P151240	*Fam150a*	Family with sequence similarity 150, member A	620393	Mm.388992	GO:0008150, GO:0003674, GO:0005575	1.07 ± 0.62	-0.80 ± 0.11	3.64
A_52_P577232	*Gucy2e*	Guanylate cyclase 2e	14919		GO:0016849, GO:0005886, GO:0004383, GO:0005525, GO:0005524, GO:0016829, GO:0042802, GO:0035556, GO:0004672, GO:0000166, GO:0016020, GO:0006182, GO:0006468, GO:0016021, GO:0009190, GO:0007601, GO:0050896, GO:0016772	1.06 ± 0.41	-0.80 ± 0.09	3.63
A_55_P2005794	*Gm9271*	Predicted gene 9271	668618			1.06 ± 0.56	-0.80 ± 0.76	3.62
A_66_P125200	*4933428C19Rik*	RIKEN cDNA 4933428C19 gene	71293	Mm.399556		1.06 ± 0.18	-0.79 ± 0.11	3.61
A_52_P1972						1.06 ± 0.86	-0.79 ± 0.68	3.61
A_65_P02079				Mm.27625		1.06 ± 0.75	-0.79 ± 0.40	3.61
A_55_P2542833	*Gm30178*	Predicted gene, 30178	102631991			1.06 ± 0.46	-0.79 ± 0.78	3.60
A_66_P109095	*Gm34481*	Predicted gene, 34481	102637750	Mm.99741		1.06 ± 0.74	-0.79 ± 0.13	3.60
A_51_P394441	*Olfr67*	Olfactory receptor 67	18368	Mm.377108	GO:0050911, GO:0007608, GO:0004984, GO:0007186, GO:0016021	1.06 ± 0.37	-0.79 ± 0.39	3.60
A_55_P2601116	*Dscr3*	Down syndrome critical region gene 3	13185		GO:0005622, GO:0008565, GO:0005623, GO:0005634, GO:0006886	1.05 ± 0.22	-0.79 ± 0.13	3.59
A_30_P01033428						1.05 ± 0.78	-0.79 ± 0.56	3.59
A_66_P130543	*Defb30*	Defensin beta 30	73670	Mm.82875	GO:0008150, GO:0003674, GO:0009986, GO:0045087, GO:0006952, GO:0005576, GO:0005575, GO:0042742	1.05 ± 0.11	-0.79 ± 0.78	3.59
A_30_P01025962						1.05 ± 0.30	-0.79 ± 0.05	3.57
A_55_P2770483	*Gm13715*	Predicted gene 13715	102640825	Mm.365850		1.05 ± 0.37	-0.79 ± 0.14	3.56
A_51_P184991	*Wdr64*	WD repeat domain 64	75820	Mm.50361	GO:0008150, GO:0003674, GO:0005575	1.05 ± 0.47	-0.79 ± 0.19	3.56
A_51_P293862	*Gnb3*	Guanine nucleotide binding protein (G protein), beta 3	14695	Mm.68889	GO:0043005, GO:0007165, GO:0051020, GO:0007186, GO:0005834, GO:0003924, GO:0030507, GO:0004871, GO:0044297, GO:0030425, GO:0070062	1.03 ± 0.31	-0.78 ± 0.75	3.51
A_55_P1972001	*Gm324*	Predicted gene 324	224876		GO:0008150, GO:0003674, GO:0005575	1.03 ± 0.44	-0.78 ± 0.74	3.50
A_66_P121312	*Olfr1251*	Olfactory receptor 1251	259145	Mm.376637	GO:0050911, GO:0007608, GO:0050907, GO:0004984, GO:0007186, GO:0016021, GO:0004888	1.03 ± 0.20	-0.78 ± 0.09	3.50
A_52_P546676	*Csnka2ip*	Casein kinase 2, alpha prime interacting protein	224291	Mm.246580	GO:0005515, GO:0008150, GO:0005634	1.03 ± 0.91	-0.77 ± 0.13	3.50
A_52_P559066	*Aim1l*	Absent in melanoma 1-like	230806	Mm.240080	GO:0008150, GO:0003674, GO:0005575	1.03 ± 0.46	-0.77 ± 0.39	3.50

A_55_P1987615	*Fam186a*	Family with sequence similarity 186, member A	380973		GO:0008150, GO:0003674, GO:0005575	1.03 ± 0.19	-0.77 ± 0.74	3.48
A_52_P640323	*Olfr68*	Olfactory receptor 68	18369	Mm.377109	GO:0050911, GO:0007608, GO:0004984, GO:0007186, GO:0016021	1.02 ± 0.51	-0.77 ± 0.73	3.46
A_55_P1997027	*Gm33101*	Predicted gene, 33101	102635875	Mm.421353		1.02 ± 0.55	-0.77 ± 0.09	3.45
A_52_P456448	*Ccdc81*	Coiled-coil domain containing 81	70884	Mm.252823	GO:0008150, GO:0003674, GO:0005813	1.02 ± 0.03	-0.76 ± 0.23	3.45
A_51_P202596	*Sh3tc2*	SH3 domain and tetratricopeptide repeats 2	225608	Mm.262320	GO:0005515, GO:0005886, GO:0022011, GO:0033157, GO:0031410, GO:0055037, GO:0032287	1.02 ± 0.79	-0.76 ± 0.31	3.43
A_51_P394847	*Gm11346*	X-linked lymphocyte-regulated 5 pseudogene	76024	Mm.440710		1.02 ± 0.11	-0.76 ± 0.70	3.43
A_30_P01020095						1.01 ± 0.15	-0.76 ± 0.04	3.42
A_55_P2243237	*AU015621*	Expressed sequence AU015621	102975	Mm.375584		1.01 ± 0.33	-0.76 ± 0.06	3.42
A_30_P01024290						1.01 ± 0.49	-0.76 ± 0.60	3.41
A_55_P2077901	*Cd2*	CD2 antigen	12481	Mm.22842	GO:0005515, GO:0043621, GO:0003823, GO:0042803, GO:0016337, GO:0001948, GO:0005911, GO:0004872, GO:0034113, GO:0007155, GO:0046658, GO:2000484, GO:0030971, GO:0009986, GO:0019901, GO:0032760, GO:0005102, GO:0005576, GO:0043234, GO:0042110, GO:0016020, GO:0009898, GO:0045121, GO:0016021, GO:0009897	1.01 ± 0.19	-0.76 ± 0.66	3.40
A_51_P172777	*Kcnc4*	Potassium voltage gated channel, Shaw-related subfamily, member 4	99738		GO:0005251, GO:0005244, GO:0031594, GO:0046928, GO:0005267, GO:0055085, GO:0016020, GO:0005249, GO:0008076, GO:0006810, GO:0051260, GO:0043679, GO:0034765, GO:0016021, GO:0005216, GO:0006813, GO:0071805, GO:0006811	1.01 ± 0.70	-0.76 ± 0.72	3.40
A_55_P2123626	*Zp1*	Zone pellucida glycoprotein 1	22786	Mm.24767	GO:0031012, GO:0007338, GO:0005794, GO:0016020, GO:0005886, GO:0005578, GO:0005576, GO:0016021, GO:0030141	1.01 ± 0.26	-0.76 ± 0.11	3.40
A_52_P156314	*Esr2*	Estrogen receptor 2 (beta)	13983	Mm.2561	GO:0005515, GO:0003700, GO:0006366, GO:2000252, GO:0050680, GO:0003707, GO:0043523, GO:0032993, GO:0042127, GO:0060548, GO:0007611, GO:0043025, GO:0001662, GO:0070374, GO:0004879, GO:0005496, GO:0045742, GO:0060766, GO:0000122, GO:0000978, GO:0005929, GO:0007420, GO:0008270, GO:2000378, GO:0019899, GO:0045893, GO:0043005, GO:0060065, GO:0048471, GO:0008285, GO:0005886, GO:0060068, GO:0042562, GO:0030284, GO:0005634, GO:0042975, GO:0046872, GO:0008289, GO:0005739, GO:0034056, GO:0051091, GO:0005737, GO:0001541, GO:0030518, GO:0048521, GO:0008144, GO:0045944, GO:0043401, GO:0043204, GO:0001046, GO:0006355, GO:0043065, GO:0060743, GO:0060740, GO:0048662, GO:0033574, GO:0003677, GO:0006351, GO:0043565, GO:0001077, GO:0060011, GO:0001764, GO:0008628, GO:0030520	1.01 ± 0.41	-0.75 ± 0.11	3.39
A_66_P123020	*Gm40918*	Predicted gene, 40918	105245469	Mm.247416		1.01 ± 0.15	-0.75 ± 0.09	3.39
A_30_P01020173						1.00 ± 0.25	-0.75 ± 0.20	3.38
A_52_P249798	*Rnase1*	Ribonuclease, RNase A family, 1 (pancreatic)	19752	Mm.235538	GO:0004518, GO:0004519, GO:0016787, GO:0005576, GO:0004522, GO:0003676, GO:0070062	1.00 ± 0.39	-0.75 ± 0.09	3.36
A_55_P1998943	*Oas1a*	2'-5' oligoadenylate synthetase 1A	246730	Mm.14301	GO:0005515, GO:0003725, GO:0005737, GO:0001730, GO:0048525, GO:0008270, GO:0005634	1.00 ± 0.83	-0.75 ± 0.70	3.35
A_66_P131023	*Htr3a*	5-hydroxytryptamine (serotonin) receptor 3A	15561	Mm.4831	GO:0005232, GO:0030424, GO:0005230, GO:0030054, GO:0005886, GO:0045202, GO:0051378, GO:0007210, GO:0004993, GO:0005737, GO:0016020, GO:0045211, GO:0005249, GO:0043025, GO:0006810, GO:0034765, GO:0016021, GO:0071805, GO:0006812, GO:0006811, GO:0015276	1.00 ± 0.67	-0.75 ± 0.16	3.35
A_55_P2900363	*Hlf*	Hepatic leukemia factor	217082	Mm.158903	GO:0043565, GO:0006355, GO:0003700, GO:0005634, GO:0048511, GO:0035914, GO:0003677, GO:0006351	1.00 ± 0.42	-0.75 ± 0.14	3.35
A_55_P2404439	*4930545L23Rik*	RIKEN cDNA 4930545L23 gene	78805	Mm.78702		0.99 ± 0.97	-0.74 ± 0.07	3.32
A_66_P102688	*Nfatc2ip*	Nuclear factor of activated T cells, cytoplasmic, calcineurin dependent 2 interacting protein	18020	Mm.354948	GO:0005515, GO:0005737, GO:0045944, GO:0005634, GO:0001816	0.99 ± 0.43	-0.74 ± 0.35	3.31
A_52_P463235	*Ankrd33b*	Ankyrin repeat domain 33B	67434	Mm.102470	GO:0008150, GO:0003674, GO:0005575, GO:0003677	0.99 ± 0.16	-0.74 ± 0.17	3.31
A_66_P117960	*Xrcc6bp1*	XRCC6 binding protein 1	68876	Mm.276769	GO:0008150, GO:0003674, GO:0016787, GO:0006508, GO:0008233, GO:0005575, GO:0004222, GO:0046872, GO:0008237	0.98 ± 0.23	-0.74 ± 0.13	3.29
A_55_P1979191	*Tbata*	Thymus, brain and testes associated	65971	Mm.30227	GO:0005515, GO:0005737, GO:0048515, GO:0007275, GO:0005634, GO:0007283, GO:0030154, GO:0005829	0.98 ± 0.16	-0.73 ± 0.09	3.28
A_55_P2569993						0.98 ± 0.22	-0.73 ± 0.09	3.27
A_55_P2926436	*Nyap2*	Neuronal tyrosine-phophorylated phosphoinositide 3-kinase adaptor 2	241134	Mm.313904	GO:0005622, GO:0005515, GO:0048812, GO:0014065	0.98 ± 0.31	-0.73 ± 0.20	3.27

A_51_P253984	*Pcp4*	Purkinje cell protein 4	18546	Mm.5023	GO:0043005, GO:0030424, GO:0005516, GO:0043524, GO:0005737, GO:0003723, GO:0010976, GO:0005883, GO:0005634, GO:0006469, GO:0033603, GO:0005829	0.98 ± 0.85	-0.73 ± 0.53	3.27
A_55_P2038372	*Acot6*	Acyl-CoA thioesterase 6	217700	Mm.49245	GO:0052689, GO:0047617, GO:0006637, GO:0016787, GO:0044466, GO:0034869, GO:0034946, GO:0016289, GO:0034843, GO:0016790, GO:0005777	0.97 ± 0.09	-0.73 ± 0.66	3.24
A_66_P118241				Mm.387727		0.97 ± 0.44	-0.73 ± 0.58	3.24
A_51_P115601				Mm.359393		0.97 ± 0.76	-0.73 ± 0.11	3.24
A_30_P01031824						0.97 ± 0.59	-0.73 ± 0.11	3.24
A_66_P108128						0.97 ± 0.35	-0.73 ± 0.34	3.23
A_55_P1957173	*D14Ertd670e*	DNA segment, Chr 14, ERATO Doi 670, expressed	52512	Mm.483998		0.97 ± 0.78	-0.73 ± 0.46	3.23
A_52_P1083725				Mm.392259		0.97 ± 0.37	-0.72 ± 0.08	3.22
A_51_P492266	*Spint4*	Serine protease inhibitor, Kunitz type 4	78239	Mm.160060	GO:0008150, GO:0003674, GO:0004867, GO:0010466, GO:0005576, GO:0005575, GO:0030414	0.96 ± 0.44	-0.72 ± 0.11	3.22
A_55_P1954468	*Gm5072*	Predicted gene 5072	278167	Mm.473682	GO:0008150, GO:0003674, GO:0005575	0.96 ± 0.51	-0.72 ± 0.68	3.20
A_55_P2122150	*Adgrl3*	Adhesion G protein-coupled receptor L3	319387	Mm.273631	GO:0051965, GO:0007165, GO:0016020, GO:0007186, GO:0005886, GO:0004930, GO:0030246, GO:0016021, GO:0004871, GO:0007166, GO:0004888	0.96 ± 0.77	-0.72 ± 0.17	3.19
A_30_P01031216						0.96 ± 0.20	-0.72 ± 0.44	3.19
A_66_P106280	*4831407H17Rik*	RIKEN cDNA 4831407H17 gene	77710	Mm.410773		0.95 ± 0.82	-0.72 ± 0.33	3.18
A_30_P01022825						0.95 ± 0.26	-0.71 ± 0.14	3.17
A_66_P101413				Mm.442668		0.95 ± 0.42	-0.71 ± 0.30	3.17
A_52_P476086	*Nxf3*	Nuclear RNA export factor 3	245610	Mm.336244	GO:0005622, GO:0005515, GO:0005737, GO:0006406, GO:0016973, GO:0005623, GO:0042272, GO:0005654, GO:0005634, GO:0003729	0.95 ± 0.47	-0.71 ± 0.18	3.17
A_55_P1968643	*Vmn1r173*	Vomeronasal 1 receptor 173	545934	Mm.480277	GO:0008150, GO:0003674, GO:0005575	0.95 ± 0.65	-0.71 ± 0.35	3.17
A_51_P400269	*Slc38a5*	Solute carrier family 38, member 5	209837	Mm.6055	GO:0015804, GO:0015816, GO:0016020, GO:0015171, GO:0005886, GO:0005887, GO:0015175, GO:0015187, GO:0016021, GO:0003333	0.95 ± 0.56	-0.71 ± 0.28	3.16
A_52_P633560	*Nodal*	Nodal	18119	Mm.57195	GO:0005515, GO:0033505, GO:0035987, GO:0048701, GO:0005615, GO:0001947, GO:0007507, GO:0009880, GO:0042074, GO:0048646, GO:0010470, GO:0007179, GO:0048018, GO:0001944, GO:0050679, GO:0001707, GO:0022409, GO:0060136, GO:0001893, GO:0001892, GO:0007275, GO:0000122, GO:0001890, GO:0032927, GO:0048546, GO:0005160, GO:0005125, GO:0001701, GO:0001702, GO:0001842, GO:0043280, GO:0001889, GO:0010085, GO:0045165, GO:0007420, GO:0038092, GO:0045596, GO:0008083, GO:0040007, GO:0048382, GO:0055123, GO:0001831, GO:0007368, GO:0008284, GO:0007369, GO:0048327, GO:0030324, GO:0009952, GO:0002085, GO:0051091, GO:0009948, GO:0045944, GO:0043408, GO:0048729, GO:0048468, GO:0010721, GO:0035050, GO:0048859, GO:0010862, GO:0042981, GO:0005576, GO:0009966, GO:0001829, GO:0010575, GO:0045766, GO:0007399, GO:0007492, GO:0016477, GO:0019827	0.95 ± 0.44	-0.71 ± 0.68	3.16
A_65_P19639	*Gm39922*	Predicted gene, 39922	105244283	Mm.455396		0.95 ± 0.91	-0.71 ± 0.07	3.15
A_51_P193185	*Mb*	Myoglobin	17189	Mm.404074	GO:0001666, GO:0042542, GO:0005506, GO:0043353, GO:0046872, GO:0015671, GO:0007507, GO:0019825, GO:0050873, GO:0005344, GO:0006810, GO:0009725, GO:0070062, GO:0020037	0.94 ± 0.59	-0.71 ± 0.36	3.14
A_52_P119258				Mm.301942		0.94 ± 0.30	-0.71 ± 0.13	3.14
A_51_P110640	*Klk1b11*	Kallikrein 1-related peptidase b11	16613	Mm.443378	GO:0016787, GO:0004252, GO:0006508, GO:0008236, GO:0008233, GO:0005634, GO:0070062	0.94 ± 0.43	-0.70 ± 0.13	3.12
A_52_P374960	*Ostn*	Osteocrin	239790	Mm.390880	GO:0030500, GO:0003416, GO:0005102, GO:0005576, GO:0007275, GO:0005615, GO:0030154, GO:0045668, GO:0001503, GO:0005179, GO:0046325, GO:0030828, GO:0007166	0.94 ± 0.37	-0.70 ± 0.60	3.12
A_51_P433615	*Klhl6*	Kelch-like 6	239743	Mm.86699	GO:0050853, GO:0003674, GO:0005575, GO:0002467	0.93 ± 0.37	-0.70 ± 0.60	3.11
A_66_P133784						0.93 ± 0.72	-0.70 ± 0.68	3.10
A_66_P118384	*Gm31359*	Predicted gene, 31359	102633563	Mm.406600		0.93 ± 0.29	-0.70 ± 0.14	3.10
A_55_P2738242	*Hspa12a*	Heat shock protein 12A	73442	Mm.39739	GO:0008150, GO:0003674, GO:0000166, GO:0005524, GO:0070062	0.93 ± 0.15	-0.70 ± 0.40	3.09
A_51_P453569	*Olfr943*	Olfactory receptor 943	258323	Mm.223019	GO:0050911, GO:0007608, GO:0004984, GO:0007186, GO:0005887, GO:0004930, GO:0005549, GO:0016021	0.93 ± 0.19	-0.69 ± 0.16	3.07
A_55_P2640507	*Smchd1*	SMC hinge domain containing 1	74355	Mm.194450	GO:0001740, GO:0051276, GO:0005694, GO:0060821, GO:0009048, GO:0005524	0.92 ± 0.56	-0.69 ± 0.49	3.05

A_52_P474152	*Onecut3*	One cut domain, family member 3	246086	Mm.250572	GO:0005667, GO:0006355, GO:0000977, GO:0003700, GO:0006357, GO:0006366, GO:0005634, GO:0001228, GO:0003677, GO:0006351, GO:0030154, GO:0001077, GO:0045944	0.92 ± 0.63	-0.69 ± 0.12	3.04
A_66_P132222	*Mlph*	Melanophilin	171531	Mm.105208	GO:0005515, GO:0042470, GO:0048471, GO:0001725, GO:0017137, GO:0005815, GO:0032400, GO:0005623, GO:0035371, GO:0003779, GO:0030318, GO:0006605, GO:0015629, GO:0006886, GO:0046872, GO:0030425, GO:0005622, GO:0031489, GO:0051010, GO:0043473, GO:0017022, GO:0030864, GO:0070062	0.92 ± 0.48	-0.69 ± 0.35	3.04
A_55_P2491668	*Usp2*	Ubiquitin specific peptidase 2	53376	Mm.272770	GO:0006511, GO:0048471, GO:0016579, GO:0045475, GO:0004843, GO:0048512, GO:0005634, GO:0046872, GO:0042802, GO:0005737, GO:0006508, GO:0005938, GO:0043153, GO:0031625, GO:0006355, GO:0005813, GO:0030332, GO:0050821, GO:0000122, GO:0048642, GO:0048643, GO:0032922, GO:0004197, GO:0007049, GO:0045931, GO:0007517, GO:0016787, GO:0061136, GO:0008233, GO:0043161, GO:0048511, GO:0008234	0.92 ± 0.29	-0.69 ± 0.30	3.03
A_55_P2753470	*Gm30340*	Predicted gene, 30340	102632200	Mm.155822		0.91 ± 0.23	-0.69 ± 0.12	3.03
A_51_P360508	*Tmem143*	Transmembrane protein 143	70209	Mm.163766	GO:0005739, GO:0003674, GO:0016020, GO:0002244, GO:0016021	0.91 ± 0.82	-0.68 ± 0.60	3.02
A_55_P2118619	*Lasp1*	LIM and SH3 protein 1	16796		GO:0005515, GO:0015075, GO:0003779, GO:0046872, GO:0005737, GO:0034220, GO:0051015, GO:0006810, GO:0008270, GO:0030864, GO:0005856, GO:0005925, GO:0070062, GO:0006811	0.91 ± 0.47	-0.68 ± 0.19	3.00
A_52_P26626	*Fam92b*	Family with sequence similarity 92, member B	436062	Mm.334852	GO:0008150, GO:0003674, GO:0005575	0.91 ± 0.20	-0.68 ± 0.17	3.00
A_66_P127140	*C230099D08Rik*	RIKEN cDNA C230099D08 gene	320212	Mm.405575	GO:0008150, GO:0003674, GO:0005575	0.91 ± 0.47	-0.68 ± 0.07	3.00
A_55_P2811644	*Slitrk4*	SLIT and NTRK-like family, member 4	245446	Mm.101707	GO:0051965, GO:0003674, GO:0016020, GO:0016021, GO:0007409	-0.91 ± 0.49	0.68 ± 0.56	-3.00
A_55_P1968799	*Krtap1-5*	Keratin associated protein 1-5	69664	Mm.38303	GO:0008150, GO:0003674, GO:0005575	-0.91 ± 0.20	0.68 ± 0.34	-3.01
A_51_P401024	*Lrrc34*	Leucine rich repeat containing 34	71827	Mm.45373	GO:0008150, GO:0003674, GO:0005575	-0.91 ± 0.28	0.68 ± 0.55	-3.02
A_52_P440265	*LOC102631977*	Uncharacterized LOC102631977	102631977			-0.91 ± 0.16	0.68 ± 0.57	-3.02
A_52_P27396	*Nabp1*	Nucleic acid binding protein 1	109019		GO:0007093, GO:0003723, GO:0070876, GO:0005634, GO:0003676, GO:0003677, GO:0006974, GO:0005737, GO:0006281, GO:0003697, GO:0000724, GO:0010212, GO:0005654	-0.92 ± 0.65	0.69 ± 0.51	-3.04
A_55_P2182172	*Mnx1*	Motor neuron and pancreas homeobox 1	15285	Mm.103760	GO:0006355, GO:0021520, GO:0021953, GO:0005634, GO:0021675, GO:0009791, GO:0003677, GO:0006351, GO:0043565, GO:0008045, GO:0060541, GO:0048667, GO:0031016, GO:0048812, GO:0031018, GO:0030182, GO:0060539, GO:0021904, GO:0001764	-0.92 ± 0.21	0.69 ± 0.62	-3.04
A_66_P115220	*Gm5922*	Predicted gene 5922	546166	Mm.423960		-0.92 ± 0.12	0.69 ± 0.62	-3.06
A_55_P1980060						-0.93 ± 0.45	0.70 ± 0.57	-3.08
A_51_P113195	*Upk1b*	Uroplakin 1B	22268	Mm.130793	GO:0016324, GO:0016020, GO:0030855, GO:0016021, GO:0070062	-0.93 ± 0.25	0.70 ± 0.70	-3.09
A_51_P264825	*Lag3*	Lymphocyte-activation gene 3	16768	Mm.4528	GO:0045085, GO:0042289, GO:0016020, GO:0050868, GO:0045954, GO:0016021, GO:0009897, GO:0007166, GO:0004888	-0.93 ± 0.25	0.70 ± 0.64	-3.10
A_55_P2416762	*1700099I09Rik*	RIKEN cDNA 1700099I09 gene	76624	Mm.426384		-0.94 ± 0.17	0.70 ± 0.57	-3.11
A_55_P2717229	*Pou2f1*	POU domain, class 2, transcription factor 1	18986		GO:0005515, GO:0005667, GO:0030910, GO:0000976, GO:0006355, GO:0003700, GO:0060235, GO:0005102, GO:0005634, GO:0003677, GO:0006351, GO:0043231, GO:0043565, GO:0045944, GO:0003682, GO:0005654, GO:0045892	-0.94 ± 0.90	0.71 ± 0.38	-3.14
A_66_P132133				Mm.127748		-0.94 ± 0.27	0.71 ± 0.40	-3.14
A_55_P1989140	*Olfr194*	Olfactory receptor 194	433031	Mm.377953	GO:0050911, GO:0007608, GO:0004984, GO:0007186, GO:0005887, GO:0004930, GO:0005549, GO:0016021	-0.94 ± 0.13	0.71 ± 0.69	-3.14
A_55_P2903199	*Serpinb9*	Serine (or cysteine) peptidase inhibitor, clade B, member 9	20723	Mm.272569	GO:0005515, GO:0002438, GO:0043066, GO:0033668, GO:0070233, GO:0004867, GO:0006915, GO:0005634, GO:0002020, GO:0005615, GO:0005829, GO:0010951, GO:0010628, GO:0005622, GO:0009617, GO:0005737, GO:0006955, GO:0016020, GO:0001913, GO:0043154, GO:0043027, GO:0042742, GO:0070062, GO:0042270	-0.94 ± 0.21	0.71 ± 0.29	-3.14
A_52_P809876	*2010320O07Rik*	RIKEN cDNA 2010320O07 gene	69881			-0.95 ± 0.10	0.71 ± 0.59	-3.15
A_55_P2833266	*Gm34483*	Predicted gene, 34483	102637752	Mm.417210		-0.95 ± 0.93	0.71 ± 0.45	-3.15
A_52_P604164	*Ptprd*	Protein tyrosine phosphatase, receptor type, D	19266	Mm.184021	GO:0005515, GO:0005102, GO:0046426, GO:0016791, GO:0097105, GO:0006470, GO:0016020, GO:0016787, GO:0050775, GO:0016311, GO:0030182, GO:0050776, GO:0004721, GO:0016021, GO:0050839, GO:0004725, GO:0007157	-0.95 ± 0.53	0.72 ± 0.28	-3.18

A_66_P138795	*Cit*	Citron	12704	Mm.426282	GO:0005515, GO:0017124, GO:0005886, GO:0048699, GO:0007283, GO:0015629, GO:0046872, GO:0030154, GO:0035556, GO:0000910, GO:0005737, GO:0000166, GO:0016301, GO:0000086, GO:0097110, GO:0043025, GO:0007030, GO:0007091, GO:0016772, GO:0005773, GO:0032467, GO:0031985, GO:0007275, GO:0030165, GO:0005524, GO:0001726, GO:0016358, GO:0007399, GO:0016310, GO:0000070, GO:0007049, GO:0007067, GO:0004672, GO:0045665, GO:0016020, GO:0004674, GO:0008064, GO:0050774, GO:0017048, GO:0006468, GO:0016740, GO:0051301	-0.96 ± 0.43	0.72 ± 0.57	-3.19
A_51_P440155	*Gm9931*	Predicted gene 9931	791353	Mm.484430	GO:0008150, GO:0003674, GO:0005575	-0.96 ± 0.36	0.72 ± 0.71	-3.20
A_51_P496735	*Grid1*	Glutamate receptor, ionotropic, delta 1	14803	Mm.483550	GO:0005515, GO:0008328, GO:0030054, GO:0005886, GO:0005234, GO:0035249, GO:0045202, GO:0030425, GO:0035235, GO:0034220, GO:0016020, GO:0045211, GO:0035176, GO:0004970, GO:0006810, GO:0004872, GO:0016021, GO:0005216, GO:0070062, GO:0006811	-0.96 ± 0.45	0.72 ± 0.64	-3.20
A_55_P2742033	*Aqp4*	Aquaporin 4	11829	Mm.489568	GO:0005215, GO:0005886, GO:0032715, GO:0007605, GO:0032691, GO:0034219, GO:0009992, GO:0070295, GO:0016323, GO:0030104, GO:0005737, GO:0060354, GO:0034220, GO:0009925, GO:0005911, GO:0015250, GO:0015793, GO:0006810, GO:0051260, GO:0006833, GO:0015254, GO:0071333, GO:0031253, GO:0005623, GO:0030315, GO:0015670, GO:0055085, GO:0010574, GO:0042383, GO:0043234, GO:0071346, GO:0016020, GO:0005887, GO:0015288, GO:0016021, GO:0009897	-0.96 ± 0.14	0.72 ± 0.42	-3.21
A_52_P633300	*Morc1*	Microrchidia 1	17450	Mm.250060	GO:0003674, GO:0008270, GO:0007275, GO:0005634, GO:0007283, GO:0046872, GO:0030154	-0.96 ± 0.12	0.72 ± 0.59	-3.21
A_51_P109258	*Cys1*	Cystin 1	12879	Mm.52265	GO:0001191, GO:0005515, GO:0048839, GO:0006366, GO:0005886, GO:0001822, GO:0005930, GO:0005634, GO:0042995, GO:0000122, GO:0000978, GO:0043433, GO:0036064, GO:0005829, GO:0005929, GO:0001077, GO:0005737, GO:0016020, GO:0045944, GO:0003682, GO:0045121, GO:0005856, GO:0070062	-0.96 ± 0.64	0.72 ± 0.61	-3.21
A_55_P2715347	*Bub1*	Budding uninhibited by benzimidazoles 1 homolog (S, cerevisiae)	12235		GO:0005634, GO:0009790, GO:0005737, GO:0007059, GO:0000166, GO:0016301, GO:0071173, GO:0071174, GO:0016772, GO:0000780, GO:0005694, GO:0051983, GO:0006915, GO:2001244, GO:0005524, GO:0016310, GO:0007049, GO:0007067, GO:0004672, GO:0000942, GO:0016020, GO:0004674, GO:0000777, GO:0000776, GO:0005654, GO:0006468, GO:0000775, GO:0007063, GO:0016740, GO:0051301	-0.97 ± 0.12	0.73 ± 0.53	-3.23
A_55_P1989474	*Chrna3*	Cholinergic receptor, nicotinic, alpha polypeptide 3	110834	Mm.63569	GO:0005515, GO:0005230, GO:0030054, GO:0005886, GO:0045202, GO:0007268, GO:0006940, GO:0060084, GO:0051291, GO:0014056, GO:0030425, GO:0007171, GO:0006937, GO:0045211, GO:0008144, GO:0015464, GO:0042391, GO:0043025, GO:0042166, GO:0006810, GO:0007626, GO:0006812, GO:0006811, GO:0046982, GO:0042493, GO:0060079, GO:0014069, GO:0005892, GO:0007271, GO:0007399, GO:0048814, GO:0035095, GO:0007165, GO:0016020, GO:0016021, GO:0005216, GO:0004889	-0.97 ± 0.52	0.73 ± 0.70	-3.24
A_55_P2713735	*Elk1*	ELK1, member of ETS oncogene family	13712		GO:0003700, GO:0006366, GO:0000981, GO:0005634, GO:0071480, GO:0030154, GO:0030425, GO:0005739, GO:0005737, GO:0071396, GO:0003690, GO:0045944, GO:0043025, GO:0009416, GO:0043679, GO:0006355, GO:0006357, GO:0001047, GO:0000978, GO:0003677, GO:0006351, GO:0043565, GO:0001077, GO:0003682, GO:0005654, GO:0045893	-0.97 ± 0.07	0.73 ± 0.66	-3.24
A_55_P2796481	*Ptprr*	Protein tyrosine phosphatase, receptor type, R	19279	Mm.336316	GO:0005515, GO:0019901, GO:0005886, GO:0005615, GO:0035335, GO:0016791, GO:0005737, GO:0006470, GO:0016020, GO:0016787, GO:0016311, GO:0070373, GO:0004721, GO:0016021, GO:0038128, GO:0004725, GO:0010633	-0.97 ± 0.25	0.73 ± 0.69	-3.25
A_55_P2726778				Mm.293024		-0.97 ± 0.42	0.73 ± 0.69	-3.25
A_55_P2419299	*1110006E14Rik*	RIKEN cDNA 1110006E14 gene	76286	Mm.393530		-0.97 ± 0.39	0.73 ± 0.69	-3.26
A_51_P230537	*Ccdc114*	Coiled-coil domain containing 114	211535	Mm.227539	GO:0005929, GO:0003674, GO:0036157, GO:0036158, GO:0042995	-0.97 ± 0.48	0.73 ± 0.44	-3.26
A_55_P2196797				Mm.443236		-0.97 ± 0.46	0.73 ± 0.72	-3.26
A_51_P147213	*3300002I08Rik*	RIKEN cDNA 3300002I08 gene	69277	Mm.272470	GO:0008150, GO:0003674, GO:0005575	-0.98 ± 0.60	0.73 ± 0.35	-3.27
A_52_P301579	*Tppp2*	Tubulin polymerization-promoting protein family member 2	219038	Mm.296425	GO:0008150, GO:0003674, GO:0005737, GO:0005575	-0.98 ± 0.58	0.73 ± 0.73	-3.28
A_55_P2742543	*0610031O16Rik*	RIKEN cDNA 0610031O16 gene	68369	Mm.441136		-0.98 ± 0.77	0.74 ± 0.65	-3.30

A_52_P677159				Mm.48841		-0.99 ± 0.37	0.74 ± 0.68	-3.31
A_66_P107886	*Sphkap*	SPHK1 interactor, AKAP domain containing	77629	Mm.154303	GO:0005739, GO:0008150, GO:0030018, GO:0005737, GO:0010738, GO:0051018, GO:0070062	-0.99 ± 0.23	0.74 ± 0.62	-3.33
A_55_P2385917	*G370062D05Rik*	RIKEN cDNA G370062D05 gene	100038490	Mm.97885		-1.00 ± 0.10	0.75 ± 0.73	-3.35
A_66_P103933				Mm.487112		-1.00 ± 0.07	0.75 ± 0.64	-3.38
A_66_P135016	*1700044K03Rik*	RIKEN cDNA 1700044K03 gene	67321	Mm.245802		-1.00 ± 0.41	0.75 ± 0.73	-3.38
A_55_P1963175	*Lctl*	Lactase-like	235435	Mm.436581	GO:0005903, GO:0016020, GO:0005975, GO:0005783, GO:0016021, GO:0004553	-1.01 ± 0.11	0.75 ± 0.72	-3.39
A_52_P198561	*Tmem30c*	Transmembrane protein 30C	71027	Mm.158563	GO:0008150, GO:0003674, GO:0016020, GO:0016021, GO:0005575	-1.02 ± 0.30	0.77 ± 0.66	-3.45
A_30_P01021802						-1.02 ± 0.18	0.77 ± 0.66	-3.45
A_55_P1954231	*Lrtm2*	Leucine-rich repeats and transmembrane domains 2	211187	Mm.121498	GO:0051965, GO:0003674, GO:0016020, GO:0016021, GO:0005575	-1.03 ± 0.20	0.77 ± 0.53	-3.47
A_66_P134849	*1700045H11Rik*	RIKEN cDNA 1700045H11 gene	73348	Mm.396218		-1.03 ± 0.11	0.77 ± 0.64	-3.48
A_51_P337125	*Inpp5d*	Inositol polyphosphate-5-phosphatase D	16331	Mm.15105	GO:0005515, GO:0017124, GO:0008285, GO:0016064, GO:0005886, GO:0005884, GO:0045579, GO:0051425, GO:0030487, GO:0045409, GO:0034594, GO:0008340, GO:0045779, GO:0005829, GO:0005622, GO:0035556, GO:0005737, GO:0045656, GO:0045659, GO:0030853, GO:0009968, GO:0043065, GO:0050869, GO:0006915, GO:0030889, GO:0045671, GO:0004445, GO:0050777, GO:0016020, GO:0016787, GO:0045648, GO:0016311, GO:0046856, GO:0030863, GO:0045621, GO:0002376	-1.03 ± 0.20	0.77 ± 0.60	-3.48
A_52_P344098	*Pde7b*	Phosphodiesterase 7B	29863		GO:0007165, GO:0008081, GO:0016787, GO:0004115, GO:0004114, GO:0046872	-1.04 ± 0.66	0.78 ± 0.61	-3.51
A_52_P1187949	*Trim5*	Tripartite motif-containing 5	667823	Mm.482375	GO:0005737, GO:0004842, GO:0002221, GO:0016567, GO:0000932, GO:0042803, GO:0042802, GO:0008329	-1.04 ± 0.30	0.78 ± 0.56	-3.52
A_51_P191726	*Efcab6*	EF-hand calcium binding domain 6	77627	Mm.196288	GO:0008150, GO:0006355, GO:0005509, GO:0005634, GO:0005575, GO:0046872, GO:0006351	-1.04 ± 0.43	0.78 ± 0.25	-3.54
A_55_P1990603	*Olfr1448*	Olfactory receptor 1448	258696	Mm.223131	GO:0050911, GO:0007608, GO:0004984, GO:0007186, GO:0005887, GO:0004930, GO:0005549, GO:0016021	-1.04 ± 0.25	0.78 ± 0.78	-3.54
A_55_P2181753	*Bdh1*	3-hydroxybutyrate dehydrogenase, type 1	71911	Mm.293470	GO:0005739, GO:0005737, GO:0005543, GO:0008152, GO:0016491, GO:0005743, GO:0003858, GO:0005654, GO:0003824, GO:0055114	-1.05 ± 0.23	0.79 ± 0.78	-3.58
A_51_P470205	*Mob3b*	MOB kinase activator 3B	214944	Mm.32968	GO:0008150, GO:0003674, GO:0005575, GO:0046872	-1.06 ± 0.43	0.79 ± 0.77	-3.60
A_51_P275435	*2310007B03Rik*	RIKEN cDNA 2310007B03 gene	71874	Mm.158766	GO:0008150, GO:0003674, GO:0005575	-1.07 ± 0.46	0.81 ± 0.53	-3.68
A_52_P59778	*Gm9725*	Predicted gene 9725	70793	Mm.475068	GO:0008150, GO:0003674, GO:0005575	-1.08 ± 0.65	0.81 ± 0.74	-3.70
A_66_P118644	*Basp1*	Brain abundant, membrane attached signal protein 1	70350	Mm.29586	GO:0005515, GO:0072112, GO:0030054, GO:0005886, GO:0044212, GO:0003714, GO:0019904, GO:0005634, GO:0042995, GO:0031982, GO:0005737, GO:0008180, GO:0016020, GO:0016607, GO:0045892, GO:0070062	-1.08 ± 0.34	0.81 ± 0.20	-3.70
A_55_P2561592	*Gm33802*	Predicted gene, 33802	102636846			-1.09 ± 0.50	0.82 ± 0.78	-3.74
A_55_P1957555	*Cypt9*	Cysteine-rich perinuclear theca 9	664724	Mm.437189	GO:0008150, GO:0003674, GO:0005634	-1.09 ± 0.35	0.82 ± 0.68	-3.77
A_51_P169671	*Reg3b*	Regenerating islet-derived 3 beta	18489	Mm.2553	GO:0008284, GO:0050830, GO:0045177, GO:0005576, GO:0006953, GO:0005615, GO:0042588, GO:0043234, GO:0050829, GO:0005737, GO:0043524, GO:0006954, GO:0060548, GO:0001934, GO:0030246	-1.10 ± 0.38	0.82 ± 0.76	-3.78
A_55_P2192617	*AI854905*	Expressed sequence AI854905	99757	Mm.39952		-1.10 ± 0.09	0.82 ± 0.66	-3.79
A_65_P12673				Mm.461431		-1.10 ± 0.41	0.83 ± 0.20	-3.80
A_52_P155818	*Abca14*	ATP-binding cassette, sub-family A (ABC1), member 14	67928	Mm.158178	GO:0008150, GO:0003674, GO:0006869, GO:0042626, GO:0005575, GO:0055085, GO:0043231	-1.10 ± 0.20	0.83 ± 0.81	-3.80
A_52_P810893				Mm.412810		-1.11 ± 0.26	0.83 ± 0.82	-3.83
A_51_P388412	*Cd55*	CD55 antigen	13136	Mm.101591	GO:0004857, GO:0005886, GO:0009986, GO:0031225, GO:0046718, GO:0043086, GO:0030450, GO:0005829, GO:0008289, GO:0016324, GO:0016020, GO:0001618, GO:0045087, GO:0006958, GO:0045121, GO:0009897, GO:0070062, GO:0002376	-1.11 ± 0.36	0.83 ± 0.82	-3.85
A_55_P2924981	*Pkd2l2*	Polycystic kidney disease 2-like 2	53871	Mm.445792	GO:0005262, GO:0050982, GO:0016020, GO:0005509, GO:0070588, GO:0006810, GO:0016021, GO:0006811	-1.12 ± 0.20	0.84 ± 0.83	-3.87
A_55_P2279685	*2900076A07Rik*	RIKEN cDNA 2900076A07 gene	100504421	Mm.217071	GO:0008150, GO:0003674, GO:0005575	-1.12 ± 0.31	0.84 ± 0.75	-3.91
A_51_P370163	*Ctr9*	Ctr9, Paf1/RNA polymerase II complex component, homolog (S, cerevisiae)	22083	Mm.255858	GO:0005515, GO:2000653, GO:0033523, GO:0001832, GO:0080182, GO:0005634, GO:1900364, GO:0001711, GO:0005622, GO:0045638, GO:0045944, GO:2001162, GO:0070102, GO:0016055, GO:0042169, GO:0006355, GO:0000993, GO:0001826, GO:0035327, GO:0000122, GO:0001829, GO:0007259, GO:0006351, GO:0071222, GO:0051571, GO:0019827, GO:0032968, GO:0016593, GO:2001168, GO:0010390	-1.12 ± 0.23	0.84 ± 0.80	-3.91

A_55_P2609124	*Gm4894*	Predicted gene 4894	235327	Mm.246604	GO:0008150, GO:0003674, GO:0005575	-1.14 ± 0.28	0.86 ± 0.83	-4.01
A_52_P192377	*Lgsn*	Lengsin, lens protein with glutamine synthetase domain	266744	Mm.389093	GO:0005515, GO:0006807, GO:0005886, GO:0004356, GO:0003824, GO:0006542	-1.16 ± 0.24	0.87 ± 0.71	-4.08
A_55_P2717762	*Gm5148*	Predicted gene 5148	381438	Mm.490549	GO:0008150, GO:0003674, GO:0005575	-1.17 ± 0.06	0.87 ± 0.80	-4.11
A_66_P139988	*1700109I08Rik*	RIKEN cDNA 1700109I08 gene	73591	Mm.65499		-1.17 ± 0.38	0.88 ± 0.41	-4.12
A_52_P1003430	*Cypt15*	Cysteine-rich perinuclear theca 15	78631	Mm.243738	GO:0008150, GO:0003674, GO:0005575	-1.18 ± 0.30	0.88 ± 0.63	-4.17
A_55_P2023732	*Limch1*	LIM and calponin homology domains 1	77569	Mm.374778	GO:0008150, GO:0003674, GO:0031032, GO:0008270, GO:0003779, GO:0005575, GO:0046872	-1.19 ± 0.95	0.89 ± 0.71	-4.24
A_55_P2731591	*Arhgef28*	Rho guanine nucleotide exchange factor (GEF) 28	110596	Mm.252718	GO:0005515, GO:0060052, GO:0005886, GO:0035023, GO:0003723, GO:0021955, GO:0030154, GO:0046872, GO:0005085, GO:0035556, GO:0005737, GO:0016020, GO:0005089	-1.20 ± 0.48	0.90 ± 0.40	-4.28
A_55_P2016515	*Lrrc69*	Leucine rich repeat containing 69	73314	Mm.52613	GO:0008150, GO:0003674, GO:0005575	-1.22 ± 0.26	0.91 ± 0.88	-4.38
A_55_P1979549	*Rp1*	Retinitis pigmentosa 1 (human)	19888	Mm.294263	GO:0008017, GO:0042461, GO:0035082, GO:0060041, GO:0060042, GO:0071482, GO:0042995, GO:0045494, GO:0035556, GO:0035845, GO:0005737, GO:0005856, GO:0046549, GO:0005930, GO:0005875, GO:0046548, GO:0005929, GO:0001917, GO:0046785, GO:0030030, GO:0007026, GO:0032391, GO:0007601, GO:0050896, GO:0001750	-1.25 ± 0.30	0.94 ± 0.67	-4.56
A_52_P533889	*4933427E13Rik*	RIKEN cDNA 4933427E13 gene	71234	Mm.158627		-1.25 ± 0.27	0.94 ± 0.90	-4.57
A_52_P467861	*Urad*	Ureidoimidazoline (2-oxo-4-hydroxy-4-carboxy-5) decarboxylase	231903	Mm.291286	GO:0019428, GO:0006144, GO:0016831, GO:0005575, GO:0016829, GO:0005777	-1.26 ± 0.13	0.94 ± 0.88	-4.61
A_51_P407786	*Ly6h*	Lymphocyte antigen 6 complex, locus H	23934	Mm.22154	GO:0016020, GO:0005886, GO:0031225	-1.27 ± 0.52	0.95 ± 0.90	-4.66
A_55_P2095558	*1600027J07Rik*	RIKEN cDNA 1600027J07 gene	69794	Mm.396139	GO:0008150, GO:0003674, GO:0005575	-1.29 ± 1.06	0.97 ± 0.93	-4.80
A_55_P2157730	*Gpr158*	G protein-coupled receptor 158	241263	Mm.166647	GO:0005515, GO:0007165, GO:0008277, GO:0016020, GO:0007186, GO:0005886, GO:0004930, GO:0016021, GO:0004871, GO:0072659	-1.30 ± 0.08	0.98 ± 0.81	-4.85
A_65_P19430	*Gm19818*	Predicted gene, 19818	100503660	Mm.382007		-1.32 ± 0.34	0.99 ± 0.96	-4.98
A_66_P105234	*Olfr1049*	Olfactory receptor 1049	259018	Mm.223405	GO:0050911, GO:0007608, GO:0004984, GO:0007186, GO:0005887, GO:0004930, GO:0005549, GO:0016021	-1.34 ± 0.37	1.00 ± 0.87	-5.05
A_66_P105645	*4930405E02Rik*	RIKEN cDNA 4930405E02 gene	78150	Mm.243806		-1.34 ± 0.05	1.01 ± 0.87	-5.09
A_55_P2100982	*Olfr1219*	Olfactory receptor 1219	258901	Mm.377742	GO:0050911, GO:0007608, GO:0050907, GO:0004984, GO:0007186, GO:0016021, GO:0004888	-1.37 ± 0.08	1.02 ± 0.27	-5.24
A_51_P104681	*Krtap6-5*	keratin associated protein 6-5	68484	Mm.46209	GO:0005882, GO:0008150, GO:0003674, GO:0005575	-1.37 ± 0.14	1.03 ± 0.95	-5.30
A_66_P133814	*Slc10a1*	Solute carrier family 10 (sodium/bile acid cotransporter family), member 1	20493	Mm.486361	GO:0016323, GO:0016020, GO:0005887, GO:0015721, GO:0008508, GO:0006810, GO:0035725, GO:0016021, GO:0015125, GO:0006814, GO:0015293, GO:0006811	-1.45 ± 0.28	1.09 ± 0.94	-5.82
A_55_P2062469	*Col12a1*	Collagen, type XII, alpha 1	12816	Mm.3819	GO:0031012, GO:0005581, GO:0005578, GO:0005595, GO:0005576, GO:0007155, GO:0005615, GO:0070062	-2.05 ± 0.66	1.53 ± 1.50	-11.96
A_55_P2508648	*Gm19757*	Predicted gene, 19757	100503536	Mm.366960		-2.11 ± 0.17	1.58 ± 1.41	-12.87

[a]Probe Name on the SurePrint G3 Mouse GE v2 8x60K Microarray (Agilent Microarray Design ID: ID 074809, Agilent Technologies)

[b]Entrez Gene ID: stable identifiers for genes and other loci for a subset of model organisms on the National Center for Biotechnology Information databases (https://www,ncbi,nlm,nih,gov/gene)

[c]UniGene ID: identifierNCBI database of the transcriptome (https://www,ncbi,nlm,nih,gov/unigene)

[d]Gen Ontology (GO): GO defines concepts/classes used to describe gene function, and relationships between molecular function, cellular component and biological process,

[e]Fold Change (FC): FC was calculated according to the following formula (Leonhardt et al, 2004): if $\log_2(FC) > 0$, $FC = 2^{(\log2(FC))}$; if $\log_2(FC) < 0$, $FC=(-1)x2^{-(\log2(FC))}$, Positive and negative values of FC showed up-regulated or down-regulated genes on Technological microcapsules, respectively,
